# Zonisamide nanodiamonds for brain targeting: A comprehensive study utilising in silico, in vitro, in vivo, and molecular investigation for successful nose-to-brain delivery for epilepsy management

**DOI:** 10.1007/s13346-025-01904-x

**Published:** 2025-07-05

**Authors:** Nihal Mohamed Elmahdy Elsayyad, Omar A. Elkady, Mohamed M. Swidan, Hassan M. Rashed, Tamer M. Sakr, Amr M. Abdelhamid, Mai A. Zaafan, Hanan M. El-Laithy

**Affiliations:** 1 Department of Pharmaceutics and Industrial Pharmacy, Faculty of Pharmacy, October University for Modern Sciences and Arts (MSA), P.O Box 12451, Giza, Egypt; 2 Labeled Compounds Department, Hot Labs Center, Egyptian Atomic Energy Authority, P.O. Box 13759, Cairo, Egypt; 3 Radioactive Isotopes and Generator Department, Hot Labs Center, Egyptian Atomic Energy Authority, P.O. Box 13759, Cairo, Egypt; 4 Department of Biochemistry and Molecular Biology, Faculty of Pharmacy, October for Modern Sciences and Arts University (MSA), P.O. Box 12451, Giza, Egypt; 5 Department of Pharmacology and Toxicology, Faculty of Pharmacy, October University for Modern Sciences and Arts (MSA), P.O. Box 12451, Giza, Egypt; 6 Department of Pharmaceutics and Industrial Pharmacy, Faculty of Pharmacy, Cairo University, Kasr El-Aini Street, P.O. Box 11562, Cairo, Egypt

**Keywords:** Neuronal networks, Molecular modelling, Zonisamide, Nanodiamonds, Nose-to-brain miR-199, Temporal lobe epilepsy model

## Abstract

The blood–brain barrier (BBB) is a stringent barrier that restricts the successful brain delivery of polar neurotherapeutics molecules. One such molecule is Zonisamide (ZNS), a hydrophilic centrally acting anti-epileptic drug. This study aims to overcome the poor ZNS BBB permeability using the nose-to-brain (NTB) carbon-based biocompatible nanodiamonds (ND) delivery system to deliver ZNS directly to the brain, bypassing the BBB, thereby enhancing its efficacy and reducing systemic side effects associated with oral ZNS currently available formulation in clinical practice. Intranasal (IN) ND-ZNS formulations were optimised using an Artificial neuronal network (ANN) and assessed for particle size (PS), zeta potential, loading efficiency (%LE), morphology, and in vitro release. The optimum radiolabelled ND-ZNS complex F1 biodistribution in different organs and its pharmacokinetics were compared to oral and IN-free ZNS in mice. Temporal lobe epilepsy (TLE) model in rats was used to compare the anti-epileptic activity of IN ND-ZNS F1 to IN free ZNS by assessing brain activity, epileptic biomarkers such as (brain neuronal specific enolase (NSE), neurofilament light polypeptide (NEFL), and matrix metallopeptidase-9 (MMP-9)), hippocampal histopathology and the modulatory effect on epigenetic miR-199/SIRT-1 and PVT-1/BDNF pathways. Optimized ND-ZNS complex F1 consists of a ZNS:ND ratio of 1:2 and sonicated for 5 min exhibited the least PS (193.7 ± 19.3 nm), adequate %LE (87.1 ± 9.2%) similar to ANN predictions, with a biphasic in vitro release profile of ZNS, beneficial for both acute and chronic epilepsy treatment. The IN delivery of ND-ZNS complex F1 showed preferential higher in vivo brain uptake with minimal systemic exposure linked with higher brain/blood ratio and significant (*p* ≤ 0.05) overall enhanced pharmacokinetics expressed by C_max_ and AUC _(0-120min)_ when compared to oral and IN free ZNS_._ Moreover, the TLE model confirmed the improved anti-epileptic activity of F1 compared to IN-free ZNS regarding brain activity and hippocampal histopathology, significant suppression of serum NSE, NEFL, MMP-9 levels, miR-199/SIRT-1 pathway, and normalization of PVT-1/BDNF pathway. Therefore, ND used in this study could be a novel, promising carrier to target ZNS directly to the brain via the IN route for effective epilepsy management with less drug dosing and the least systemic side effects.

**Graphical Abstract:**

**Figure:**
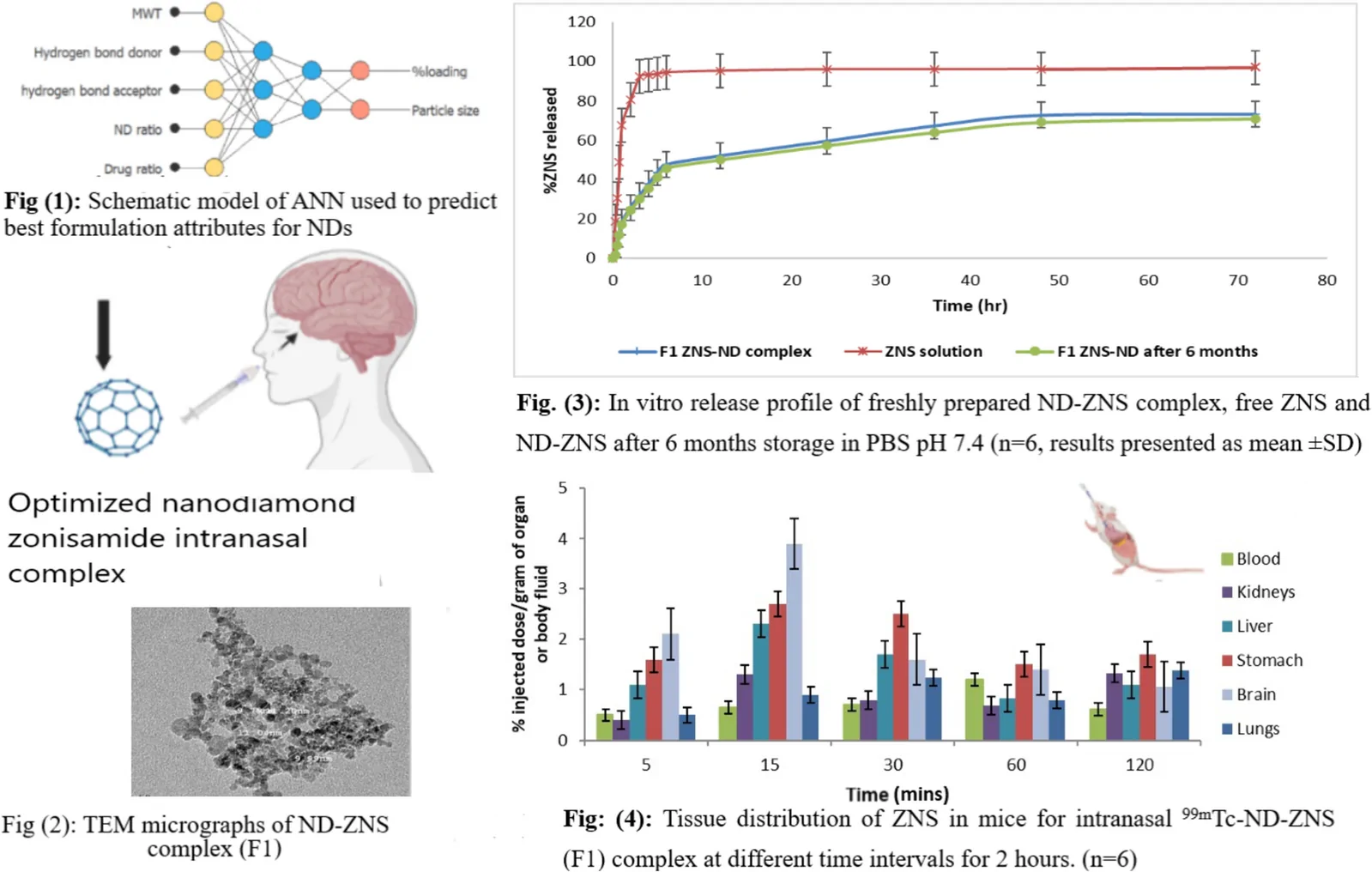

## Introduction

Despite recent advancements in drug delivery systems for the treatment of neurological disorders, However, direct and effective drug delivery to the brain remains a significant challenge due to the complex anatomical and physiological nature of the blood-brain barrier (BBB) that selectively limits the entry of drugs into the brain [1]. The BBB tight cellular junctions restrict the diffusion of polar molecules from the blood through the capillary endothelial cells and into the CNS, hindering their transport via passive delivery and decreasing their clinical efficacy in the brain [2].

One such polar drug is Zonisamide (ZNS), a sulphonamide-based anti-epileptic drug effective against focal frontal and temporal lobes seizures in adults and children [3]. ZNS exerts its effect on the brain by inhibiting the neuronal voltage-dependent sodium and T-type calcium channels, modulating the dopaminergic system, and accelerating gamma-amino-butyric acid [4]. However, ZNS reported a 28% Oldendorf Brain Uptake Index following its systemic absorption, signifying low distribution in the brain [5]. ZNS's low distribution in the brain can be attributed to multiple factors, mainly its hydrophilic nature combined with its binding to erythrocytes and intercellular proteins, accounting for its inability to pass BBB through passive transport [5]. Although the commercial prevalence of oral ZNS formulation, the oral clinical efficiency is limited by (i) the extensive hepatic first-pass metabolism, which inactivates the drug by cytochrome P450 (CYP) 3A4 (ii) as a carboanhydrase inhibitor, ZNS reported to induce nephrolithiasis with the formation of kidney stones [3]. Therefore, developing an alternative route to enhance ZNS brain uptake with an improved adverse effect profile would compromise its clinical use as a first-line drug.

Nose to Brain (NTB) delivery can eliminate problems associated with the delivery of centrally acting drugs as it circumvents BBB completely and allows rapid drug delivery through the olfactory and trigeminal nerve pathways located at the roof of the nasal cavity directly to the brain [6]. NTB also surpasses the conventional oral route as it bypasses hepatic first-pass metabolism and avoids systemic absorption through the blood, thereby overcoming drug-protein binding and achieving higher and faster drug concentration in the brain [7]. However, NTB suffers from rapid drug elimination from the nasal cavity due to mucociliary clearance [8].

Thus, the need for a suitable carrier to improve ZNS IN delivery and increase its passage through the olfactory pathway to achieve successful ZNS brain delivery with minimal systemic absorption is highly recommended and challenging due to the poor IN absorption of hydrophilic ZNS molecule [9]. Notably, the use of a hydrophobic nanocarrier with particle size (PS) below 300nm could assure adequate olfactory delivery by facilitating the movement of particles through the endothelial cells to the olfactory neurons via endocytosis thereby avoiding nasal clearance and achieving efficient NTB delivery [10]. Carbon-based nanodiamonds (NDs), with their intrinsic hydrophobicity, biocompatibility, and chemical inertness, can be a promising carrier for successful NTB delivery. Their proven safety on brain cells and nasal mucosa, as well as their nanometre size and round surface, can offer an additional unique advantage in NTB delivery as it enhances endocytosis by enabling the particles to interact more with olfactory and nasal mucosa, requiring less energy to stimulate endocytosis thus leading to better direct internalization of ND-ZNS to the brain where ZNS can be released at its site of action [11]. Moreover, NDs have higher biocompatibility than other carbon-based nanomaterials, like carbon nanotubes and nanographene, which have been linked to toxicity in previous research. Furthermore, they are not dispersed in water, making employing them in nanoformulations difficult. On the contrary, the surface electrostatic potential of ND, which draws water to the surface, enables drug molecule adsorption [12]. In addition, these unique properties of NDs offer several advantages over other nanoparticles, such as liposomes and other polymeric nanoparticles, such as higher stability due to their diamond lattice structure, which can withstand harsh physiological conditions, unlike liposomes, which are prone to leakage and degradation in vivo [13, 14]. Moreover, the high surface area of NDs allows for high drug loading capacity and easily loads multiple molecules and ligands due to their abundant functional groups, thus allowing for targeting and stimuli-responsive release [13]. In comparison, other nanoparticles may have low encapsulating capacity of hydrophobic drugs, such as lipid nanoparticles, or require extensive modifications, such as polymeric nanoparticles, to achieve high loading capacity [13, 15]. Also, their proven biocompatibility has surpassed other nanoparticles, which, depending on their composition, can trigger the body’s immune system [16].

To the best of our knowledge, no reported trials have adopted ND for IN application to improve NTB delivery of hydrophilic molecules. Therefore, based on the aforementioned considerations, the present study aims to explore the potential of Artificial Neural networks (ANNs) for the optimization of ZNS-loaded ND (ND-ZNS) to reach an optimal NTB ND-ZNS formulation with enhanced ZNS brain uptake and anti-epileptic efficacy. ANNs, a machine learning computer algorithm, have been used to optimize formulas by making accurate predictions by training and detecting patterns in previously published data between formulation parameters and outputs to produce accurate predictions without actual lab experimentation, thus avoiding extensive experimentation and time with the excessive cost of materials and manpower to achieve such optimization [17, 18].

The in vivo pharmacokinetics and biodistribution of the optimized ND-ZNS in the brain, blood, liver, lung, and kidney were also conducted and compared to oral ZNS, the readily available treatment in mice after radiolabelling with ^99m^Tc to assess the ability of ND-ZNS to enhance brain delivery with minimum peripheral exposure after IN administration. A third objective was to demonstrate that ND formulation could benefit the nose-to-brain uptake by comparing the anti-epileptic activity of IN ND-ZNS formulation with IN ZNS solution in a Temporal lobe epilepsy (TLE) rat model through modulation of miR-199/Sirt-1 and PVT-1/BDNF pathways. The spontaneous recurrent seizures were recorded using electroencephalogram (EEG), and epileptic biochemical markers such as neuron-specific enolase (NSE), neurofilament light polypeptide (NEFL), and matrix metallopeptidase-9 (MMP-9) were also measured as well.

## Materials and methods

### Materials

ZNS was a kind gift from Mash premiere (Cairo, Egypt). ND powder (PS<10 nm), Sodium dithionate dihydrate, and Pilocarpine hydrochloride were purchased from Sigma-Aldrich (Darmstadt, Germany). Spectra/Pore dialysis membrane (12,000–14,000 molecular weight cut-off) was purchased from Spectrum Laboratories Inc. (USA). Technetium-99m was eluted as ^99m^TcO_4_ from ^99^Mo/^99m^ Tc generator, Radioisotopes Production Facility, Egypt. Atropine sulfate was obtained from a chemical industries development company (Cairo, Egypt). Standard rat ELISA kits of Neuron-specific enolase (NSE): CAT no. NBP2-76684 and matrix metallopeptidase-9 (MMP-9): CAT no RMP900 were obtained from Novus biologicals (Colorado, USA). Standard rat ELISA kits for neurofilament light polypeptide (NEFL): CAT no. E-EL-R2536, and brain-derived neurotrophic factor (BDNF): CAT no. E-EL-R1235 was purchased from Elabscience (Texas, USA). Direct-zol RNA Miniprep Plus: CAT no. R2072 was purchased from Zymo Research Corp. (California, USA), and reverse-transcriptase kit CAT no. 12594100 was obtained from Thermo Fisher Scientific (Massachusetts, USA). Materials: Paper chromatography (PC) (Whatman International Ltd, Maidstone, Kent, UK). All other chemicals used were of analytical grade and were obtained from standard commercial suppliers. Normal male Swiss albino mice of body mass range 25–50 g were purchased from Helwan University, Egypt, to be used in the in vivo pharmacokinetic studies, while adult male Wistar albino rats (150–200g) were acquired from *Teodor Bilharis* Institute (Cairo, Egypt) for studying the in vivo anti-epileptic activity.

### Artificial neuronal network (ANN) design

**Fig. 1:**
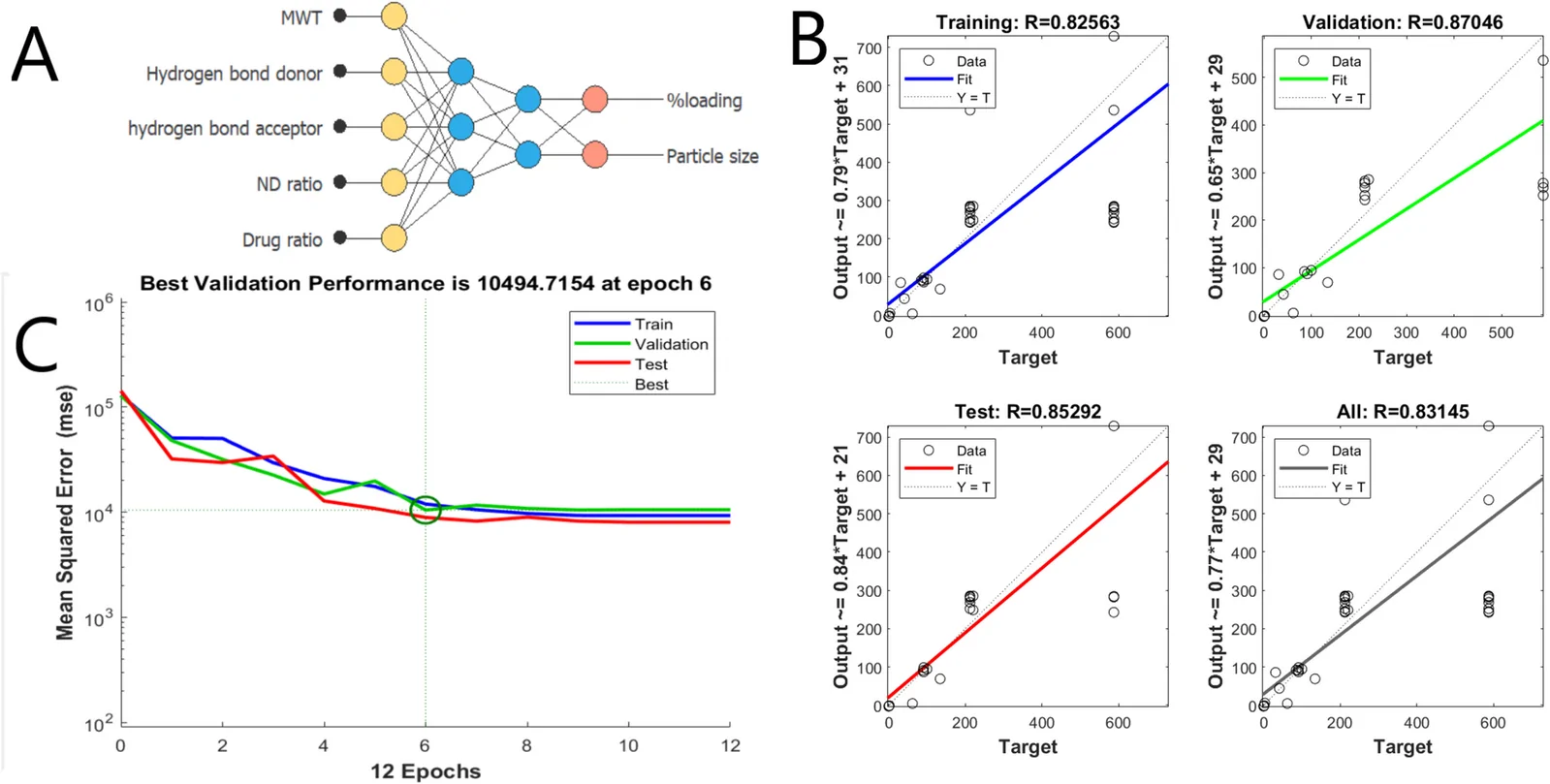
(**A**) Schematic model of ANN used to predict best formulation attributes for NDs (Drawn using Neural Designer version, V.4.2, Artelnics, Spain). (**B**) Fitting plot for ANN used for prediction of best formulation attributes for NDs (Matlab 2019ra). (**C**) Validation performance during training (mean square error plotted at different epochs)

Artificial Neuronal Network (ANN) training and validation were conducted using Neural Network Toolbox (NNTool) of MATLAB^®^ R2019a (MathWorks Inc., Natick, MA, USA) to predict the optimized formulation attributes for ZNS loading on ND, namely PS and loading efficiency (%LE). The dataset included previously published ND formulations using different drugs extracted from the Web of Science utilizing data points from previous similar research and preliminary trials conducted during this study (data not shown) [12, 19–31]. ANN was constructed through the following five basic steps: (1) Collecting data as previously described, (2) pre-processing data, (3) building the network, (4) training, and (5) testing the model performance. Additionally, feedforward networks were created for this study. Based on literature review and data mining, the ratio of drug to ND, molecular weight of the drug, number of hydrogen bond donors, and hydrogen bond acceptors in the drug molecule were selected as input factors (Fig. 1A). Historical data collected from published literature were randomly divided into training (65%), testing (20%), and validation (15%) sets. Levenberg–Marquardt algorithm was used to train ANN, and a sigmoidal function ("logsig") was used to transfer the hidden layer. The mean square error was used as the performance function. The stopping criteria for ANN training were six validation failures or 1000 epochs, whichever came first [32, 33].

The predicted data were compared with the original data set by plotting the predicted versus original values and computing the correlation coefficient for each response in the output layer. The closer the correlation coefficient values to 1, the better the predictive capability of the ANN. Several training sessions were conducted with different numbers of nodes in the hidden layer and training times to determine the optimal ANN architecture. The ANN with the closest correlation coefficient value to 1 was then selected for further prediction from hypothetical formulations. The predicted data were compared with the original data set by plotting the predicted versus original values and computing the correlation coefficient for each response in the output layer. The closer the correlation coefficient values to 1, the better the predictive capability of the ANN. The selection of optimal formulation inputs was done by the brute-force method with a step of variation 1/20 of the original input data range for each factor (ZNS molecular weight at 212.3, the number of hydrogen bond donors as one, and hydrogen bond acceptors as five as retrieved from Pubchem^®^ and ZNS and ND ratios ranging from 1 to 5). The selecting criteria were the responses of %LE of not less than 80% and PS not more than 250 nm. The determined formula was prepared, and the generated responses (%LE and PS) were measured in vitro. The predicted and real responses were compared using percentage bias between actual and predicted values calculated using Eq. (1) [34].

1 $$Percentage\;Bias\;(\%)=\frac{Actual\;Value-Predicted\;Value}{Actual\;Value}x\;100$$

### Loading of ZNS on NDs

NDs were first dispersed in deionized water, followed by sonication using a probe sonicator (Dismembrator, Fisher Scientific) for 5 minutes to form ND stock suspension of 40 mg/ml. ZNS was loaded on ND by adding 1 ml of ZNS stock solution (containing 20 mg ZNS) to 1 ml (containing 40 mg) of ND dispersion and diluted to 10 ml. The ND-ZNS dispersion underwent probe sonication for a duration of 5, 10, or 60 minutes. The composition and sonication time of all formulae are presented in Table 1.

**Table 1:** Composition and sonication times of different prepared ND-ZNS complexes as well as their respective PS, PDI and zeta potentials presented (*n* = 3, results presented as mean ± SD)

Formula	Sonication time (mins)	Composition (mg/ml)		PS (nm)	PDI	ZP (mV)	%LE
		ZNS	ND				
Blank ND	5	0	1	182.2 ± 13.8	0.21	19.5 ± 1.2	-
F1	5	1	2	193.7 ± 19.3	0.37	17.13 ± 1.7	87.1 ± 9.2
F2	10	1	2	498.6 ± 81.2	0.40	17.32 ± 2.02	79.2 ± 12.1
F3	60	1	2	2604.8 ± 501.2	0.24	17.7 ± 0.2	68.2 ± 15.3

###  Loading Efficiency (%) 

An indirect technique was utilized in determining the loading efficiency (%LE) of ZNS on ND [35, 36]. Briefly, ND-ZNS loaded suspensions were centrifuged at 12000 rpm for 1 hour (Centrifuge model 5417R, Eppendorf, Hamburg, Germany), after which the supernatant was separated, and ZNS amount was determined in supernatant spectrophotometrically at λ_max_ of 235±2nm using distilled water as a blank. The measurements were done in triplicates and calculated according to an established ZNS standard calibration curve. The %LE was calculated using Eq. (2) [37].

2 $${\%LE}=\frac{\text{Total ZNS in ZNS loaded ND }-\text{Free unbound ZNS in supernatant }}{\text{Total ZNS in ZNS loaded ND}}\mathrm{x}100$$

### Particle size and Zeta potential

The mean PS and Zeta potential (ZP) of ND-ZNSs were determined by photon correlation spectroscopy (PCS) technique using Malvern Zetasizer (Nano-ZS, Malvern Instruments, Malvern, UK). All measurements were performed at 25 ± 0.5 °C in triplicate, and results are presented as mean± standard deviation [38].

### Transmission electron microscopy (TEM)

The ND-ZNS optimized formula (F1) morphological characteristics were examined using transmission electron microscopy (JEM-1230, Jeol, Tokyo, Japan). A sample from the F1 formula was deposited on the carbon-coated copper grid (200 mesh) and negatively stained with a 1% aqueous solution of phosphotungstic acid, and the excess was drawn off using filter paper. The sample was completely dried at ambient temperature and examined using a JEM-2100 high-resolution transmission electron microscope (Jeol, Tokyo, Japan) at an acceleration voltage of 80 kV under different magnification powers [39].

### Molecular modeling

The model was based on the simplest diamond structure, Adamantane, which was used previously for modeling ND, as it has the same spatial arrangement of carbon atoms in a diamond crystal [40]. In the present work, the Adamantane structure was modified by adding carboxylic, amino, and hydroxyl groups to mimic the functional groups present on the ND structure due to the detonation process. The chemical structures of Adamantane and ZNS were obtained using PubChem^®^ in SDF format and imported into Maestro^®^ software (Maestro^®^ V.12.1.013, Schrodinger Inc, USA). Energy minimization and alignment were performed for all the investigated molecules, and possible interactions were inspected and compared [41].

### Fourier transform infrared spectroscopy (FTIR)

Fourier transform infrared spectroscopy (FTIR) was performed for ZNS, ND, and optimized ND-ZNS (F1) using an IR Affinity spectrometer (Shimadzu, Japan) over a range of 400−4000 cm−1. Two milligrams of samples were mixed with 0.1 g of potassium bromide (KBr) powder using mortar and pestle, after which the sample was pressed to a thin film before the spectra were taken. The resulting spectra were obtained and compared [42, 43].

### In vitro drug release

The in vitro release study utilized a Franz diffusion cell with a diffusion area of 1.77 cm^2^ [44]. The receptor compartment contained 7.5 ml phosphate buffer (PBS) pH 7.4, simulating the pH at the release site in the brain [45, 46], and the system was equilibrated at 37 ± 0.5 °C by a circulating water jacket. 1 ml of ND-ZNS complex F1 was filled into a dialysis bag (molecular weight cut-off: 12,000–14,000 Da) previously soaked overnight in the release medium before use, tightly tied from both ends and then loaded into the receptor compartment, which was constantly stirred at 150 rpm with a small magnetic bar. At certain time intervals (0, 0.25, 0.5, 0.75, 1, 2, 4, 8, 12, 24, 36, 48, and 72 h), 0.5 ml samples were aliquoted from the receptor compartment and replaced with equal volumes of fresh medium to maintain a constant volume. Samples were filtered using a 0.22 μm filter and analyzed spectrophotometrically as previously described. In order to understand the barrier presented by the dialysis membrane, the in vitro release study of unformulated ZNS (free drug) solution in distilled water of the same concentration (1mg/ml w/v) was carried out similarly in a separate setup, all release experiments were done in triplicates, and the release data were fitted to various release kinetic models: zero, first, Higuchi diffusion, and Hixson–Crowell and Korsmeyer–Peppas models.

### Stability study

ND-ZNS optimized formula (F1) was stored in glass containers in a refrigerator at 4 °C ± 0.5 °C for six months according to the ICH guideline (Q1A (R2) section 2.1.7.1). After storage, Particle size and LD% were re-determined and compared to the freshly prepared complex.

### In vivo pharmacokinetics and biodistribution of ND-ZNS complex

#### Radiolabelling of ZNS

ND-ZNS complex (F1) and free ZNS were radiolabelled using Technitium-99 (^99m^Tc) by adding 20 mg sodium dithionate to the 1 mg ZNS solution in phosphate buffer pH 7. Then, 100 μL of freshly eluted ^99m^Tc-pertechnetate; 99mTcO4- (200 MBq) was added to the above mixture, and the reaction mixture was vigorously shaken for 15 minutes. Radiochromatographic analysis was developed using the ascending paper chromatography (PC) technique to monitor the radiochemical purity of the ^99m^Tc-labelled compound using two distinct mobile phases; acetone was used to detect free pertechnetate, which migrated to Rf = 1, while the other component remained at Rf = 0 and a second mobile phase, comprising NH4OH, C2H5OH, and H2O (1:2:5), was used to determine the reduced hydrolyzed (R-H) 99mTc-colloid, which remained at Rf = 0, whereas ^99m^Tc-labelled compound and free pertechnetate migrated to Rf = 1 [7, 47]. Two drops of the reaction product and free 99mTcO4 were placed on two strips of Whatman PC on the origin line, 2 cm far from the base, to be developed by developing solvents. The experiments were conducted in triplicate to ensure the reproducibility and reliability of the results. As the developing process was terminated, the strip was dried, trimmed into 1 cm size pieces, and counted using the sodium iodide (NaI) γ-ray scintillation counter (A good-type NaI scintillation γ-Counter model scalar rate meter SR7 (Nuclear Enterprises Ltd., USA). The radiochemical yield percent of ^99m^Tc–ZNS was calculated using Eq. (3)

3 $$\%Radiochemical\;yield=100-\%Free\;99\mathrm{mTc}-\%colloid\;99\mathrm{mTc}$$

#### Brain uptake and biodistribution

To study the uptake of free ZNS via oral and IN routes and IN ND-ZNS complex (F1) in the brain as well as different organs, namely, brain, lungs, stomach, liver, and kidneys, male Swiss albino mice of body mass range 25-50g were used [48]. The experiment protocol was approved by the University of October for Modern Sciences and Arts (MSA) ethical committee (Pt2/Ec2/2022PD), where the principles of the Declaration of Helsinki were followed. The mice were maintained in cabins of a size that suits groups of five at room temperature with a 12 h light/dark cycle and accessibility to food and water ad libitum. The mice were divided into four groups. Mice in group 1 served as a control and received no treatments. Mice in group 2 were given ZNS orally using an oral gavage (100 μL). Mice in group 3 were injected intranasally with ^99m^Tc–ND-ZNS complex in each nostril (100 μL) with the help of a micropipette attached with a low-density polyethene tube having 0.1 mm internal diameter at the delivery site. Mice in group 4 were injected intranasally free ^99m^Tc–-ZNS similarly. The mice were held from the back in a slanted position during nasal administration of the formulations. Next, mice were anesthetized by chloroform at 5, 15, 30, 60, 120 min post-injection (*n*=6 mice/time point). Samples of fresh blood and different organs were collected, washed twice using saline solution, made free from adhering tissue/fluid, and weighed. The radioactivity in each tissue/organ was measured using a shielded well-type gamma scintillation counter. The radiopharmaceutical uptake per gram in each tissue/organ was calculated as a fraction of the administered dose. Percent injected dose per gram organ and body fluid (mean %ID per gram organ and body fluid ± SD) in a population of six mice were calculated, and the significance level was set at *p* > 0.05.

#### Pharmacokinetics and data analysis

Drug concentrations in plasma and brain were investigated based on the measured radioactivity as previously discussed. Blood samples were collected at 5, 15, 30, 60, and 120 min post-injection (*n*=6 mice/time interval). Plasma and brain pharmacokinetic parameters were calculated by non-compartmental analysis using the Microsoft Excel Pharmacokinetics add-on. The rate of drug absorption was estimated through maximum plasma/brain concentration (C_max_), time for maximum plasma/brain concentration (T_max_), the extent of drug absorption via the area under the curve from zero to time (AUC_0-t_), and the area under the curve from zero to infinity (AUC _0-∞_). The data in the form of mean values (± SD) of three determinations were statistically analyzed by applying one-way ANOVA using Minitab 17 software. Data was expressed as mean±S*.*D and analyzed by ANOVA followed by Tukey's test for multiple comparisons. Differences were considered significant when *p* was less than 0.05.

The brain-targeting potential of ZNS oral solution, free IN ZNS, and IN ND-ZNS complex was determined by utilizing the Brain/Blood ratio and logBB at time points 15 (Cmax) and 120 minutes calculated using eqs. 4 and 5, respectively:

4 $$Brain/ Blood Ratio=\frac{\text{AUC Brain}}{\text{AUC Blood}}$$

5 $$LogBB=Log\frac{\%ID\;in\;the\;Brain}{\%ID\;in\;the\;Blood}$$

### In vivo investigation of the anti-epileptic activity of intranasal ND-ZNS complex

#### Induction of temporal lobe epilepsy (TLE) in rats

Temporal lobe epilepsy (TLE) was induced in rats using the pilocarpine model according to the method of Arshad et al. [49]. Prior to the experiment, rats were housed in plastic cages at October University for Modern Science and Arts under controlled environmental conditions (temperatures of 25°±3°C and 50% humidity) with free access to water and softened standard pellet chow. The rats were injected with pilocarpine (300 mg/kg; i.p.) to induce status epilepticus (SE). Atropine (1 mg/kg; i.p.) was injected 30 minutes before pilocarpine injection to antagonize the peripheral muscarinic activity. SE was defined using the modified Racine scale by observing seizure severity categorized as stages 4 and 5. If rats did not exhibit stage 4 or 5 seizures within 30 minutes after the initial pilocarpine dose, they received a supplemental pilocarpine injection dose (30–60 mg/kg, i.p.). The SE seizures were left to last for 3 hours to induce hippocampus damage, after which diazepam (10 mg/kg; i.p.) was injected to attenuate the seizures. Lubricant ophthalmic gel was applied with a cotton swab to each eye to prevent the corneas from drying out during the seizures. The rats were hydrated with sterile Ringer's lactate injections (1 mL, i.p.) for 3 days post-SE induction. Animal welfare assessments were conducted multiple times per day for several days after the induction of SE. The Pilocarpine-inducted rats began to develop spontaneous recurrent seizures (SRS) as a characteristic of TLE within 3 weeks, after which the treatments were injected for 7 days, and the in vivo pharmacodynamic investigation was conducted [49–51]. Scoring of seizures based on the modified scale according to the method previously described where 0 indicates normal activity, 1 indicates facial automatisms, 2 indicates tail stiffening and wet-dog shakes, 3 indicates low-intensity tonic-clonic seizure marked by unilateral forelimb myoclonus, 4 indicates bilateral forelimb myoclonus and rearing, and 5 indicates bilateral fore- and hindlimb myoclonus and transient loss of postural control. Racine scoring offers a standardized way to assess seizure severity and progression, making it a valuable tool in epilepsy research, especially in rodent models. This scoring system categorizes seizures into distinct stages based on the observed behavioral manifestations, making it a valuable tool for assessing the efficacy of anti-epileptic treatments [49, 52].

### Experimental design

Rats were randomly allocated into four groups (*n* = 8). The first group was a normal control group that only received saline. TLE was inducted into the other three groups. The second group served as the TLE-positive control group and did not receive any treatment, while the other two groups (third and fourth) were treated intranasally with free ZNS and ND-ZNS complex, respectively. The treatments were administered intranasally for 7 days after the occurrence of SRS (21 days after pilocarpine injection) in a dose of 16 mg/kg/day of ZNS [9].

At the end of the experiment, SRS was monitored using a video recorder (6 hours/day) and scored according to the Racine scale [53]. In addition, the brain activity was recorded using an electroencephalogram (EEG, powerlab module, which consists of Power-lab/8sp and Animal Bio-Amplifier, Australia). The rats were anesthetized with urethane (1.5 g/kg; i.p.), and the blood samples were collected for biochemical analysis [54]. At the end of the experiment, the rats were sacrificed by cervical dislocation, and the brains were rapidly isolated to be used for biochemical investigations in the hippocampus as well as histopathological examination.

### Biochemical assays

The serum levels of neuron-specific enolase (NSE), matrix metallopeptidase-9 (MMP-9), neurofilament light polypeptide (NEFL), and brain-derived neurotrophic factor (BDNF) were measured by ELISA technique using the following standard kits CAT no. NBP2-76684, RMP900, E-EL-R2536, and E-EL-R1235, respectively, according to the manufacturer's procedures.

For quantitative RT-PCR analysis of miR-199, Sirtuin 1 (Sirt-1) and long non-coding RNA of plasmacytoma variant translocation1 (LncRNA PVT-1), RNA was isolated from the brain hippocampus tissue with direct-zol RNA Miniprep Plus (CAT no. R2072) and reverse-transcribed into cDNA with the reverse transcriptase (CAT no. 12594100) according to the manufacturer instructions. The following primer sequences were used in the current experiment: forward 5′- TATGCTCGCCTTGCTGTGGA −3′ and reverse 5′- GCTGAGTTGCTGGATTTTGTGT-3′ for *Sirt-1* gene, forward 5′- TGAGAACTGTCCTTACGTGACC-3′ and reverse 5′- AGAGCACCAAGACTGGCTCT −3′ for *LncRNA PVT-1* gene, and *GAPDH* housekeeping gene was forward 5'-CCCATCACCATCTTCCAGGAG-3'and reverse 5'- GAAGGGGCGGAGATGATGAC-3'. As regards *miRNA 199, the* primer sequence was forward 5′- GCGCCCAGTGTTCAG −3′ and reverse 5′- GTGCGAGGTCCGAGT −3′. Normalization for variation in the expression of the *miRNA 199* gene was performed versus the *U6* housekeeping gene by the ΔΔCt method. *U6* gene primer sequence was 5′- ATGACGTCTGCCTTGGAGAAC-3′and reverse 5′- AGTGCAGGGTCCGAGGTATT-3′. Quantitative reverse transcriptase PCR was performed using a Power SYBR Green PCR Master Mix (CFX96 Instrument; Bio-Rad, USA). The relative quantitation (RQ) of each target gene is quantified according to the 2-∆∆Ct method calculation.

#### Brain histopathological examination

Rats'brain tissue samples were fixed in 10% neutral buffered formalin for 72 hours. Samples were processed in serial grades of ethanol, cleared in Xylene, and then infiltrated and embedded into Paraplast tissue embedding media *(Leica Biosystems).* 5μn thick serial sagittal brain sections were cut by rotatory microtome to demonstrate hippocampal regions in different samples and mounted on glass slides. Tissue sections were stained with Hematoxylin and Eosin as a standard staining method for light microscopic examination and then examined in a blinded manner and the recorded lesions were scaled from 0–4, where, 0 indicates no lesions, 1 indicates few lesions in one examined section, 2 indicates mild lesions were focally demonstrated in some examined sections, 3 indicates moderate lesions were diffusely demonstrated in some examined sections, and 4 indicates severe lesions were diffused in all examined sections [55]. Data were obtained using a Full HD microscopic imaging system (Leica Microsystems GmbH, Germany).

### Statistical analysis

Statistical analysis was performed using Minitab^®^ software (version 17). Data was analyzed by using a one-way analysis of variance (ANOVA) followed by the Tukey-Kramer multiple comparisons test. Differences were considered significant when *p* was less than 0.05. Kruskel–Walli's test was used to analyze the scores of motor seizures and histopathological scores, followed by Dunn's multiple comparisons tests. Statistical tests of the in-vivo anti-epileptic activity were conducted using the GraphPad Prism software package, version 5 (GraphPad Software, Inc., USA).

## Results and discussion

### Artificial neuronal network

Artificial Intelligence and machine learning models offer a promising solution for overcoming time and resource constraints in scientific research through efficient historical data mining and analysis, thereby substituting numerous trial and error experiments [56]. In this study, an ANN was employed to optimize ND-ZNS through data analysis of previous literature to predict actual outcomes without the need for actual experimentation. ANN was employed due to its superior accuracy in data analysis, compared to the traditional Design of Experiments and statistical models, in addition to its ability to identify relationships between various input and output factors, leading to substantial time and cost savings in practical lab work [32, 57, 58].

After reviewing relevant literature, PS and %LE were selected as the output attributes in this study due to the criticality of PS in ensuring adequate IN delivery and to ensure maximal drug loading onto ND to be delivered to the brain. The ANN model was designed according to schematic Fig. 1A and was trained several times until optimal accuracy was achieved. Model accuracy is determined by fitting the predicted and the actual data for the training, validation, and test sets of the data and calculating the coefficient of determination (R^2^), which should have a value ranging from 0.8 to 1 for the model to be considered accurate [33]. As depicted in Fig. 1B, the model underwent successful training and validation, evidenced by the good fit between actual and predicted data, confirmed by overall R2 values above 0.8 (ranging from 0.82 to 0.87), which indicates a strong correlation between predicted and actual responses with minimal error [57]. The best validation performance was observed at 6 epochs with the least mean square error (MSE) observed, indicating successful training and validation of the model (Fig. 1C).

Consequently, the established ANN model was used to optimize the ND- ZNS formulation by setting ZNS molecular weight at 212.3, the number of hydrogen bond donors as one, and hydrogen bond acceptors as five as retrieved from Pubchem^®^ and utilizing several ZNS: ND ratios between 1 and 5 using brute-force method. ZND: ND ratio of 1:2 showed the best-predicted attributes of PS and %LE; thus, this formula was prepared in the lab, and PS and %LE were measured. As depicted in Table 2, the predicted and the measured responses of PS and %LE were comparable with a similar bias to previous similar research between actual and predicted values for PS and %LE (12.18% for PS and 4.02% for %LE) [34, 59]. These results indicate the success of ANN as an in-silico tool to correctly predict formulation responses according to the given attributes without the need for actual experimentation and unwarranted resource consumption.

**Table 2:** ANN Evaluation of Predicted vs. Actual %Drug Loading and Particle Size in ND-ZNS optimized formula based on input factors ZNS: ND ratio of 1:2, molecular weight of ZNS (212.23 g/mol), number of hydrogen bond donor groups (1) and hydrogen bond acceptor groups (5) based on ZNS chemical structure

Parameter	Predicted value	Actual value	Percent bias (%)
%LE	83.6	87.1 ± 9.2	4.02%
PS	217.3	193.7 ± 19.3	12.18%

### ZNS loading efficiency

As shown in Table 1, ZNS was successfully loaded on ND surfaces with drug loading % ranging from 68.2±15.3% to 87.1±9.2%. Due to the presence of several functional groups, the extensively charged ND surfaces formed an electrostatic interaction with ZNS, resulting in layers of ZNS covering ND surfaces. Moreover, it is rather interesting to point out the observed inverse relationship between %LE and ultrasonication time where F1 ND-ZNS complex (sonicated for 5 min) revealed the highest %LE of 87.1±9.2 followed by F2 (sonicated for 10 min) of 79.2±12.1 %LE and the least was F3 (sonicated for 60 min) with %LE of 68.2±15.3. This trend stems from the increased breakage tendency of the week hydrogen bonds by increasing the ultrasonication vibration energy, leading to decreased interaction between ZNS and ND functional groups, which account for lower %LE [60].

### Zeta potential

The ZP plays a crucial role in the stability of ND dispersion and guards against particle aggregation [61]. As shown in Table 1, blank ND dispersion revealed a positive ZP of 19.5±1.2 mV due to the presence of different functional groups on the ND surface. Moreover, the presence of sp2 carbon species forming graphene-like shells within the diamond lattice is most likely responsible for an overall net positive charge due to their protonation in aqueous media [24].

Loading of ZNS on the ND surface resulted in a slight decrease in ZP of ND-ZNS complexes to 17.1±1.7, 17.3±2.0 and 17.7±0.2 mV as shown in F1, F2, and F3, respectively, regardless of sonication time, thus confirming the successful binding of ZNS on the surface of NDs [20], which will be explored further through molecular modeling in this study. Such positively charged ND-ZNS complexes lead to enhanced bio-adhesion with the negatively charged nasal mucosa via electrostatic interaction, resulting in decreased nasal clearance and improved brain bioavailability [62].

### Particle size

**Fig. 2:**
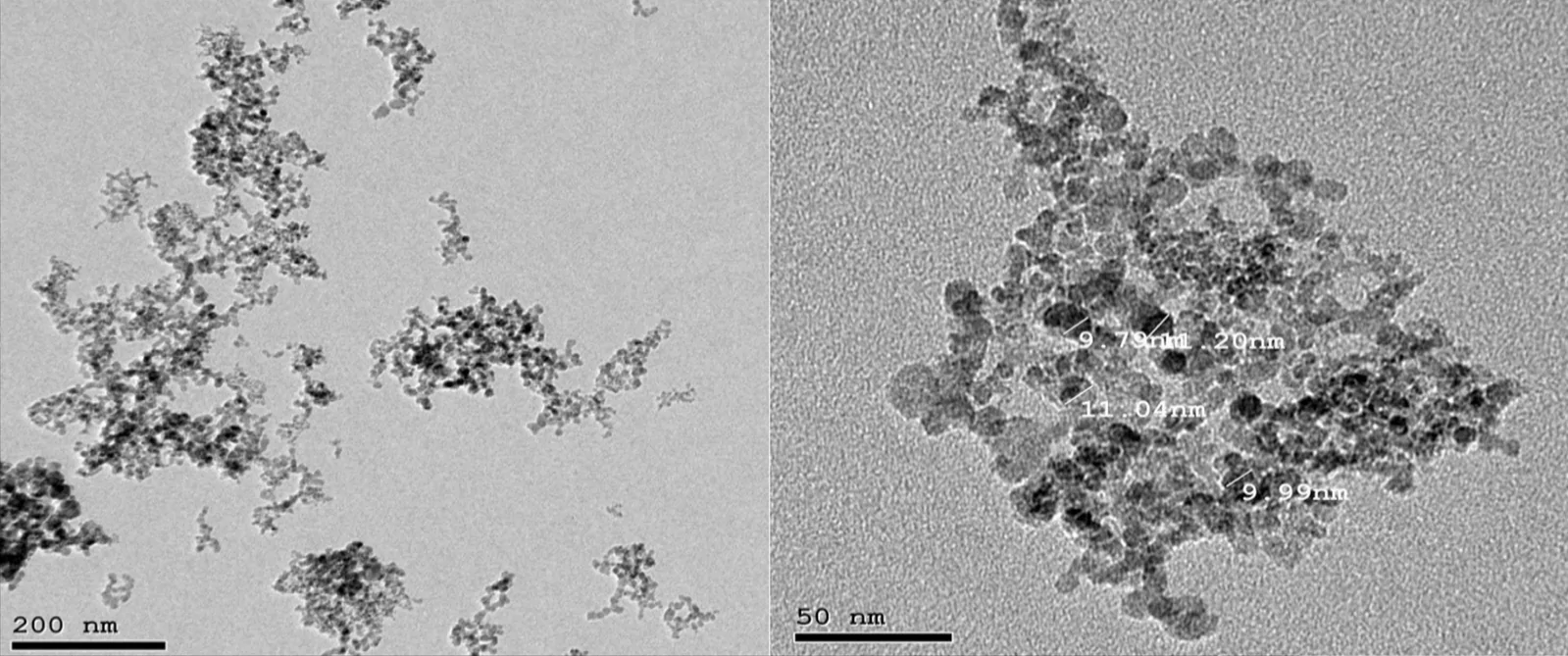
TEM micrographs of ND-ZNS complex (F1)

PS is a crucial feature for successful brain delivery through the IN route, as it should be below 300nm to ensure that the particles migrate towards the olfactory bulb through the endothelial cells via endocytosis without being trapped in the nasal mucosa [10, 62]. Although the nominal NDs are small in size (<10nm), they tend to agglomerate in aqueous media, as evidenced by the measured Z-average sizes of blank ND dispersion of 182.2±13.8 nm (Table 1) as well as TEM micrographs (Fig 2). The tendency of ND aggregation was previously described and attributed to the electrostatic interactions of several functional groups, such as hydroxyl, carboxyl, lactones, ketones, and ethers, on the surface of NDs through the detonation process upon its manufacture [63]. However, ND aggregation stops when a dynamic equilibrium is reached between the electrostatic attraction and repulsion between ND surface functional groups and water molecules in the aqueous medium. This leads to the cessation of further aggregation [64, 65].

Loading of ZNS on ND revealed an increase in the PS of ND up to 193.7±19.3 nm, which strongly indicated the successful adsorption of ZNS on ND particles to form the ND-ZNS complex [20]. Moreover, the increase in particle size of ND-ZNS can also be attributed to the decreased ZP, which leads to further aggregation of ND due to reduced charge and repulsive force. However, this effect was minimal due to the small decrease in ZP, which did not significantly affect the equilibrium between attractive and repulsive forces, resulting in only a slight increase in particle size.

Interestingly, ultrasonication proved to be the most critical process parameter affecting PS and aggregation behaviour of ND. As shown in Table 1, The particle size of ND-ZNS complexes increased significantly with prolonged sonication where PS of F1 (5 minutes sonication) <F2 (10 minutes sonication) <F3 (60 minutes sonication) with PS of 193.7±19.2 nm, 498.6±81.2 nm and 2604.6±501.2 nm, respectively. Normally, ultrasonication disaggregates coarse agglomerates to form smaller ones by producing ultrasonic waves, which causes hydrodynamic stress by collapsing cavitation bubbles, leading to agglomerate breakdown [66]. Nevertheless, these contradictory findings could be substantiated by the increase of the ultrasonication energy by increasing sonication time, leading to increasing the local pressure and temperature gradients in the ND dispersion, which causes the sonicated particles to collide and re-agglomerate forming sonication-induced aggregates [67].

Therefore, given the aforementioned results, the ND-ZNS complex (F1) possessing a %LE of 87.1±9.2%, a positive ZP of 17.1±1.7 mV, and the smallest PS of 193.7± 19.3 nm was processed for further characterization studies.

### TEM

TEM micrographs of ND-ZNS complex (F1) in Fig. 2 revealed single primary ND particles in the range of 10 nm clustered together to form larger size aggregates of approximately 200 nm. This correlates well with the PS measurements and aligns with the previous reports describing ND morphology [28]. NDs tend to aggregate due to the electrostatic interactions of several functional groups such as hydroxyl, carboxyl, lactones, ketones, and ethers on the surface of NDs through the detonation process upon its manufacture, leading to its aggregation [63]. ND aggregation stops when a dynamic equilibrium is reached between the electrostatic attraction and repulsion between ND surface functional groups and water molecules in the aqueous medium, leading to the cessation of further aggregation [64, 65].

### Molecular modeling and FTIR

**Fig. 3:**
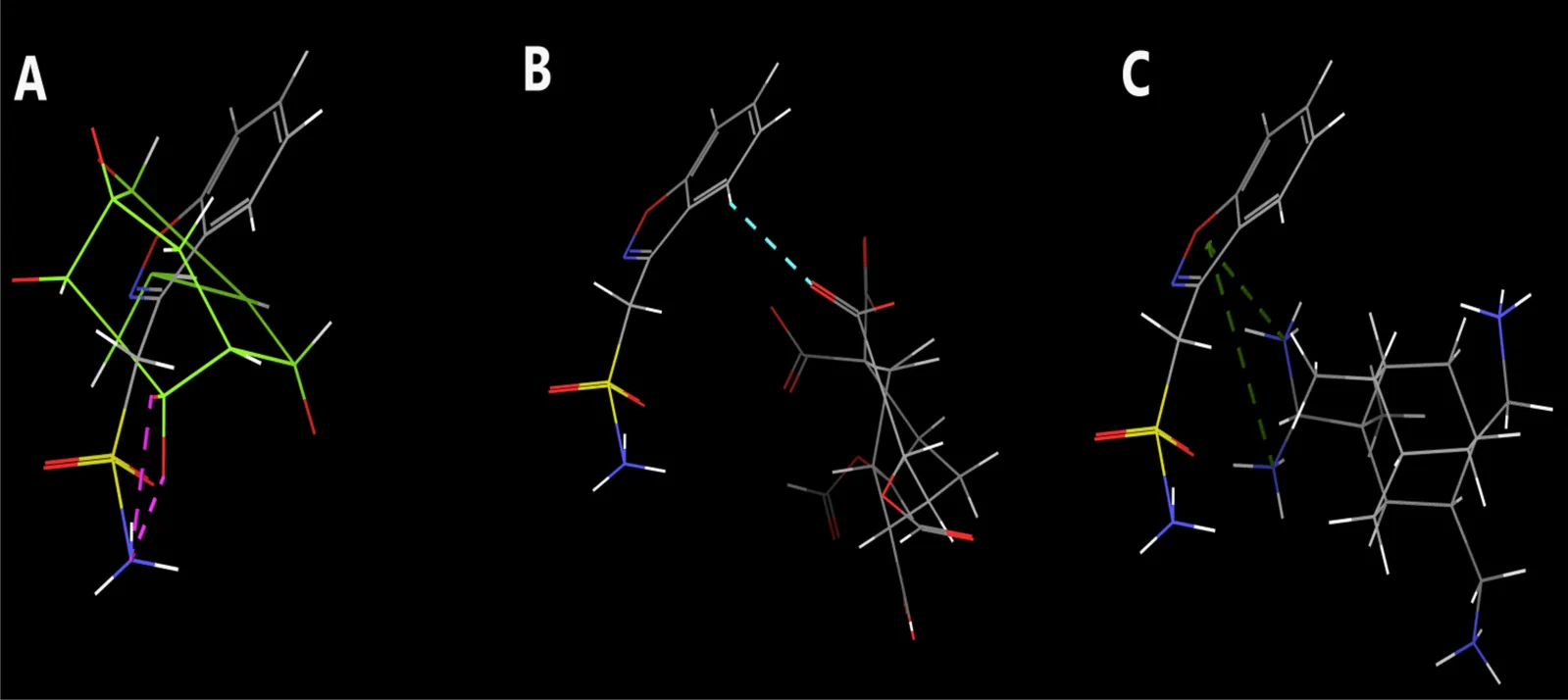
Molecular modelling of the interaction between ZNS and adamantane molecule enriched with (**A**) hydroxylic groups (-OH), (**B**) carboxylic groups (-COOH), and (**C**) amino (-NH3) groups

Molecular modeling was done through Maestro software to elucidate the mechanism of ZNS adsorption on ND and possible interactions. As shown in Fig. 3, Molecular modeling between three adamantane molecules used to mimic real ND molecules carrying –COOH, –NH3, and –OH groups were created. It is clear from Fig. 3A that the -NH^+2^ group (hydrogen bond acceptor) in ZNS formed two hydrogen interactions with -OH groups (hydrogen bond donor) present on the surface of ND. Additionally, Fig 3B revealed that C=O groups on adamantane molecules (hydrogen bond donors) formed a hydrogen bond with the aromatic ring in the ZNS structure as hydrogen bond acceptors [68]. Furthermore, Fig 3C demonstrated the Pi electrostatic interaction between the oxazole ring in ZNS as an electron-rich group with the adjacent protonated –NH^+2^ group (NH^+3^) on the ND surface.

**Fig. 4:**
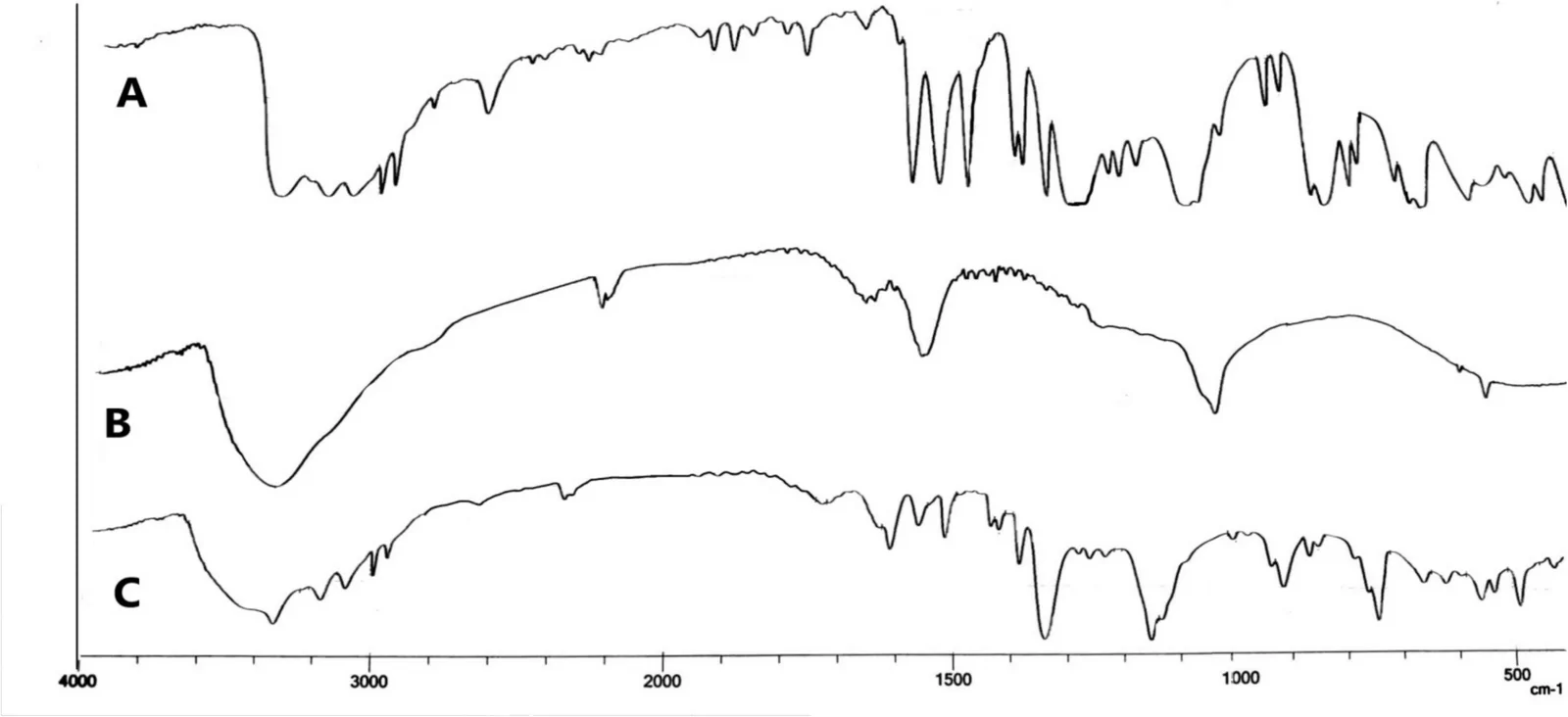
FTIR spectra of (**A**) pure ZNS, (**B**) pure ND, and (**C**) ND-ZNS complex

The obtained ND-ZNS electrostatic interactions were further confirmed by FTIR results (Fig. 4). ZNS IR spectrum with the characteristic peaks at 1610.59 cm^−1^,1564 cm^−1^ and 1516 cm^−1^ indicative of the oxazole group, peaks at 3310.87 cm^−1^, 1277 cm^−1^,1690 cm^−1^, 1383.95 cm^−1^ indicative of NH2, C-O, C=N, S=O respectively and the aromatic ring at 2989.72 cm^−1^ and 2964.32 cm^−1^ [69]. Blank ND displayed peaks corresponding to the functional groups on its surface, namely a broad peak at 3423.71 cm^−1^ corresponding to N-H stretching of amino groups which overlap with –OH stretching band, a peak at 1733.87 cm^−1^ corresponding to C=O stretching due to the presence of carboxylic groups, 1634.7 cm^−1^ corresponding to–OH groups bending and 1117.77 cm^−1^ of C-O stretching of hydroxyl groups and at 1457.25 cm^−1^ corresponding to CH bending and 619.16 cm^−1^ signifying the presence of adsorbed Cl- on its surface [70]. ND-ZNS complex FTIR spectrum revealed an overlap of both ND and ZNS characteristic peaks, thereby confirming the successful loading of ZNS on the ND surface [28]. Hydrogen bonding on the surface of ND with drugs can appear as a disappearance, shift, or decrease in the magnitude of the original peaks [70]. The electrostatic interactions were confirmed with the disappearance of 1117.77 cm^−1^ peak in ND corresponding to C=O (hydrogen bond acceptor) and the shift of ZNS aromatic ring peak from 2946.0 cm^−1^ to 3163.0 cm^−1^ confirming the hydrogen bond between both groups. Also, the shift in the frequency of 3310 cm^−1^ peaks of -NH2 to 3322.44 cm^−1^ combined with a decrease in the magnitude of –OH peak on ND confirmed the interaction between –OH and –NH2. The decrease in the magnitude of the peak at 1564.0 cm^−1^ as well as the disappearance of the C=N stretching peak at 1690 cm^−1^ that belongs to the oxazole group confirmed the interaction with the –NH^3+^ group depicted in the molecular modelling. These findings confirmed the molecular modeling data and assured that hydrogen bonding is the primary mechanism behind the high adsorption and loading capacity of ZNS on ND surfaces.

### In vitro drug release

**Fig. 5:**
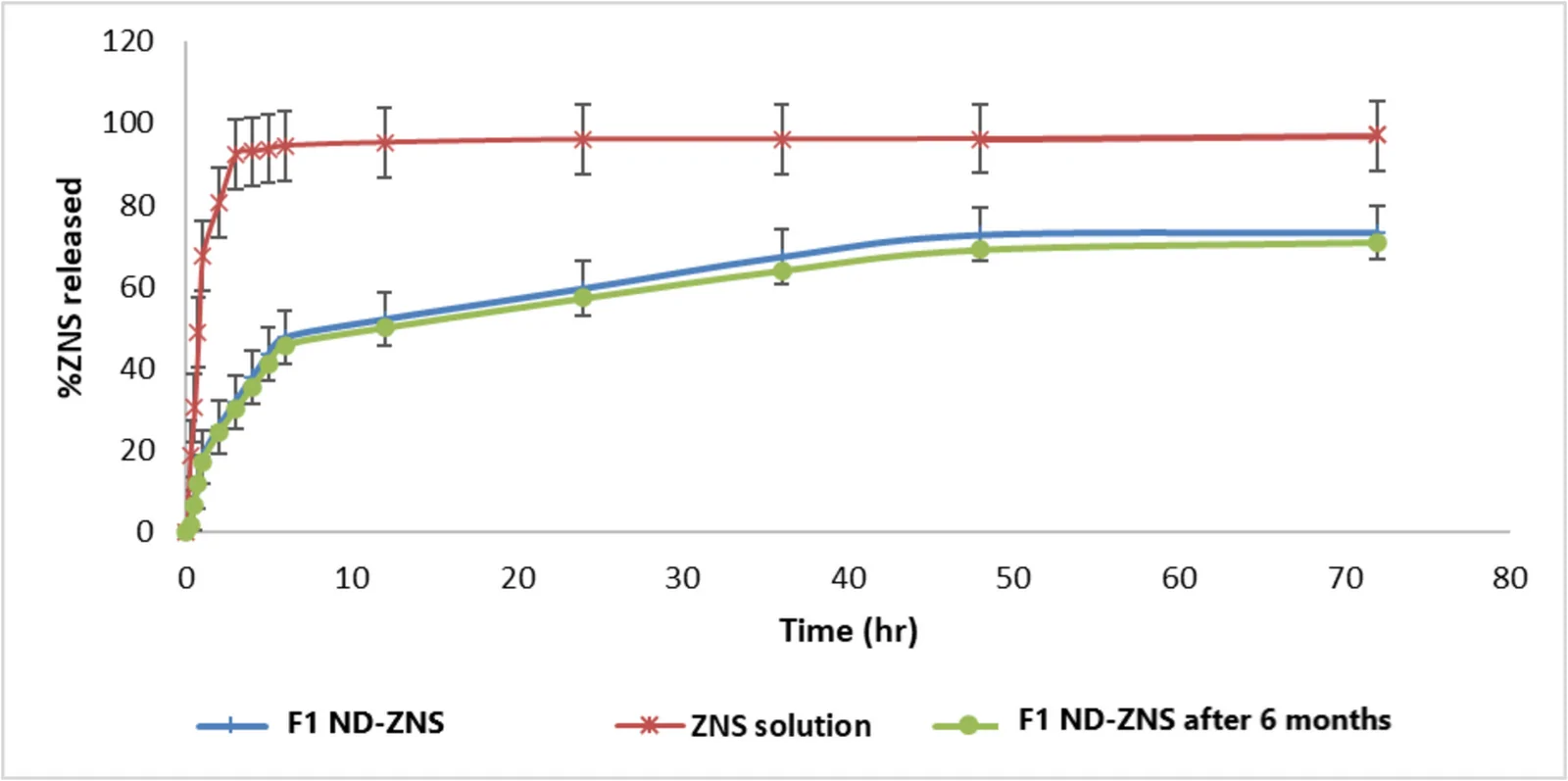
In vitro release profile of freshly prepared ND-ZNS complex, free ZNS and ND-ZNS after 6 months storage in PBS pH 7.4 at 3 ± 2 °C (*n* = 6, results presented as mean ± SD)

In vitro ZNS release studies simulating release conditions in the brain following successful NTB delivery of the ND-ZNS complexes are depicted in Fig. 5 for free ZNS and ND-ZNS complex (F1). ZNS release from ND-ZNS complex followed a biphasic in vitro release profile of ZNS, beneficial for both acute and chronic epilepsy treatment with 47.6±2.8% of ZNS released during the first 6 hours followed by a sustained pattern of 73.3±7.8% after 72 hours. This pattern was opposite to the ZNS solution, which showed an immediate rapid release of 96.9±2.1% in only 2 hours due to its high solubility. The observed biphasic profile offered by NDs is advantageous as the initial release phase can help achieve high ZNS concentration, targeting acute seizures within minutes, which is critical for halting status epilepticus or emergency episodes. Concurrently, the sustained phase would provide great therapeutic benefit if one considered the potential of higher ZNS retention for prolonged periods within the brain extracellular fluid, which helps in decreasing the brain ZNS fluctuations level, thereby reducing the frequency of the seizures, acting as a prophylactic against seizures and enhancing the patient compliance and quality of life by reducing systemic exposure and neuropsychiatric risks (e.g., depression, agitation) observed in vulnerable population [71].

The initial burst release phase can be ascribed to the interaction of water molecules with the ND's layered octahedral surfaces, which have uniquely different electrical properties on each side, resulting in water molecules re-arrangement around the ND particles, and thereby facilitating the release of the adsorbed ZNS molecules on the surface [20, 72]. In contrast, the demonstrated sustained release phase could be attributed to aggregating ND -ZNS particles together, thus burying ZNS towards the center of the aggregates and shielding it from immediate release, leading to its slow release from the ND aggregates instead [73].

The release data was fitted to various release kinetic models: zero-order, first-order, Higuchi diffusion, and Hixson–Crowell and Korsmeyer–Peppas models, and the coefficient of determination (R^2^) was calculated (Table 3). Based on the highest R^2^ value of 0.98, the release data were best fitted to the Higuchi model, indicating that the main mechanism of drug release was diffusion-controlled. Upon further fitting to the Korsmeyer-Peppas model, the diffusional exponent'n'was found to be between 0.45 and 0.89 for cylindrical geometries(n=0.51), indicative of anomalous (non-Fickian) transport where the rates of drug diffusion through pores or matrices within the nanodiamond structure and polymer relaxation associated with interactions and surface modification on ND surfaces are comparable. The near-threshold n value (0.51) suggests a slight dominance of diffusion over relaxation processes, consistent with studies on ND drug delivery systems as porous, rigid particles with limited swelling.

**Table 3:** Fitting of ZNS release kinetics fom ND-ZNS (F1) on different release models and their corresponding coefficient of determination (R^2^) and n component

Release model	*n* value	R^2^ value
Zero-Order	N/A	0.6528
First-Order	N/A	0.069
Higuchi	N/A	0.98
Hixson-Crowell	N/A	0.37
Korsmeyer-Peppas	0.51	0.97

### Stability study

The stability of the prepared ND-ZNS (F1) formula was confirmed at refrigerated conditions. After six months, the ND-ZNS complex retained its colloidal stability with a similar particle size of 213.2±18.2nm compared to the original 193.7±19.3 (*p*>0.05) of freshly prepared formula. The colloidal stability of non-functionalized ND was attributed previously to the dynamic equilibrium between the electrostatic attraction of ND particles and the simultaneous repulsion between ND surface functional groups and water molecules in the aqueous medium, which stabilizes ND aggregates against further aggregation after their initial self-assembly [64]. Moreover, the ND-ZNS complex retained its ZNS %LE of 85.8±7.1% with 71.0±7.4 % ZNS release after 72 hours in PBS pH 7.4 and similarity factor f2 > 50, *p* > 0.05 compared to 87.2±9.2% and 73.4±7.8% of freshly prepared formula, respectively. These results confirmed the stability of the ND-ZNS complex and validated its applicability as a suitable NTB dosage form.

### In vivo biodistribution and pharmacokinetics study

**Fig. 6:**
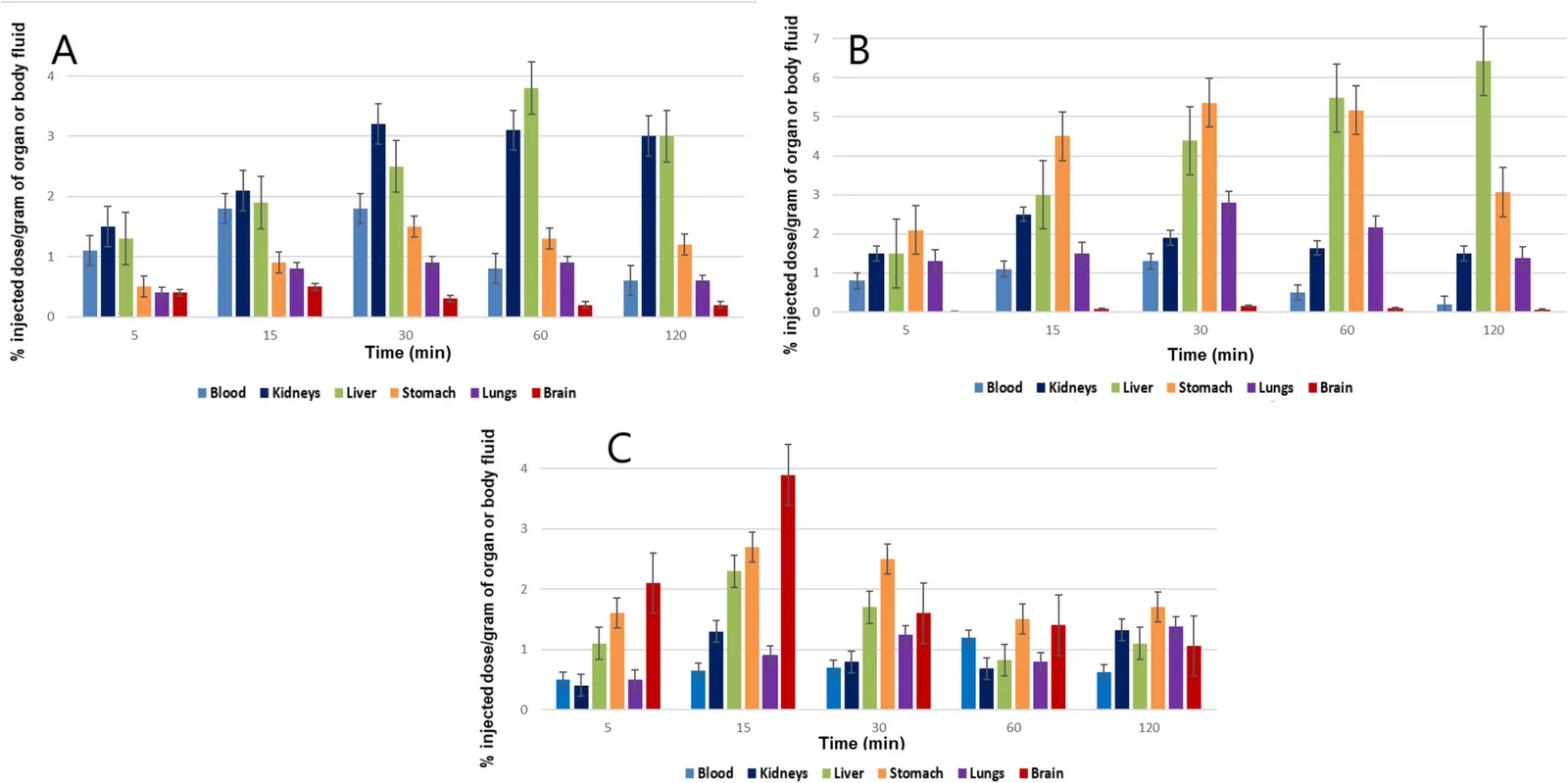
Tissue distribution of ZNS in mice for (**A**) Free ^99m^Tc-ZNS via IN route and (**B**) Free ^99m^Tc-ZNS via oral route at and (**C**).^99m^Tc-ND-ZNS (F1) complex via intranasal route at different time intervals for 2 h. (*n* = 6, results presented as mean ± SD)

**Fig. 7:**
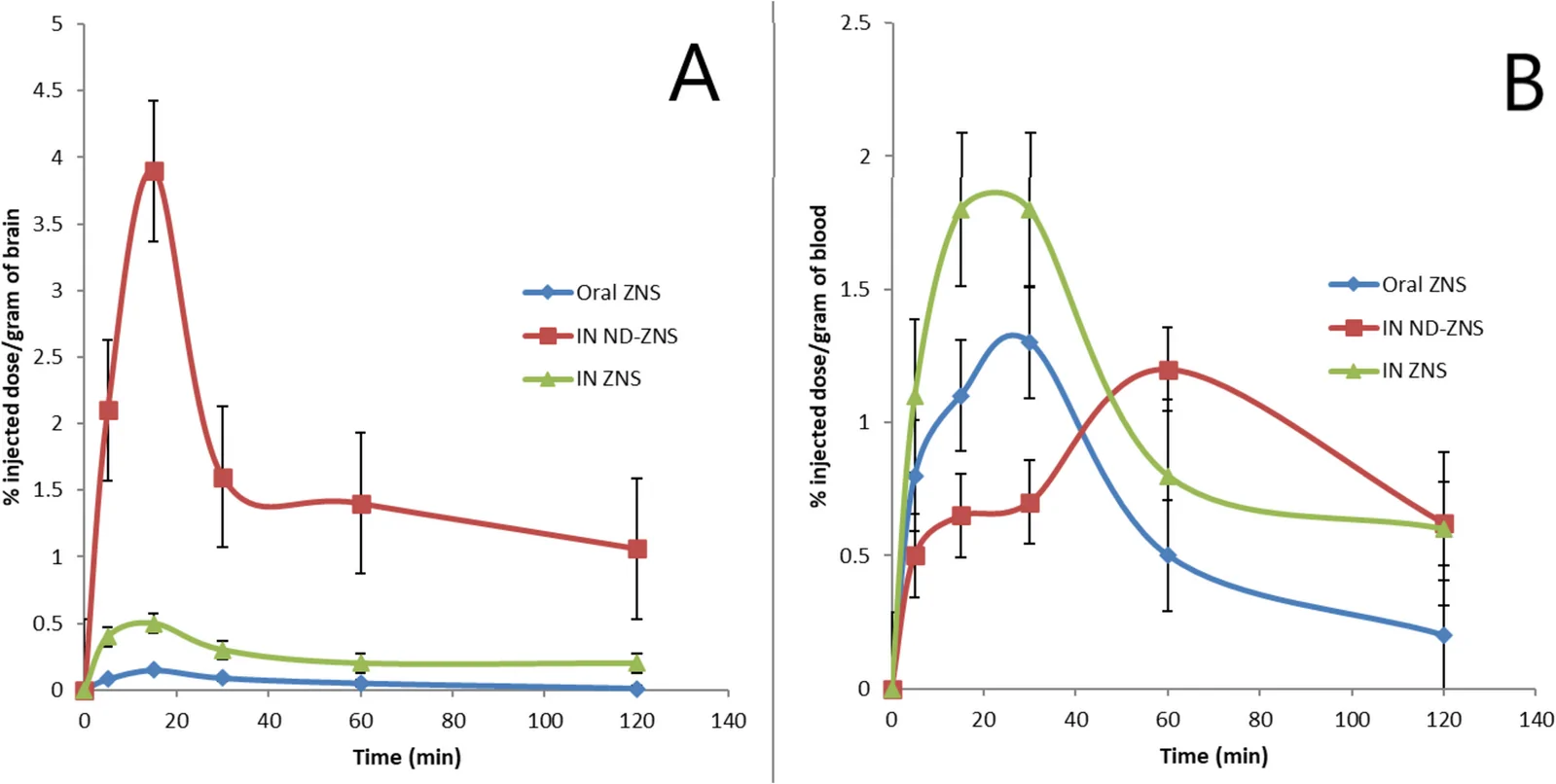
%ID/g in (**A**) brain and (**B**) blood for IN ^99m^Tc-ND-ZNS complex (F1), IN ^99m^Tc-free ZNS and oral.^99m^Tc- free ZNS in mice for 2 h (*n* = 6, results presented as mean ± SD)

^99m^Tc-free ZNS and ^99m^Tc–ND-ZNS complex (F1) were successfully prepared for the in vivo studies with 93.2±0.9% radiosynthesis yield confirmed by PC analysis. Since the present work intended to promote direct ZNS NTB targeting to minimize the systemic side effects associated with oral ZNS currently available formulation in clinical practice, the in vivo biodistribution of ZNS in different organs, namely, the brain, stomach, liver, kidney, and lungs, were assessed herein for free ZNS via the oral and IN route compared to IN ND-ZNS (F1). The pharmacokinetic behaviour in blood and brain was also studied and compared to determine ZNS circulation time, distribution, and elimination patterns in the brain to elucidate the benefits of IN delivery of ND-ZNS (F1) (Table 4 and Figs. 6 and 7).

**Table 4:** Pharmacokinetics parameters of IN ^99m^Tc-ND-ZNS complex (F1) compared to IN and oral ^99m^Tc- free ZNS after 2 h following administration to mice (*n* = 6, results presented as mean ± SD, * = significant at 0.05 level)

Organ	Intranasal ND-ZNS		Intranasal free ZNS		Oral free ZNS	
	Brain	Blood	Brain	Blood	Brain	Blood
C_max_ (%ID/g)	3.9 ± 0.1^*^	1.2 ± 0.05	0.5 ± 0.2	1.8 ± 0.7	0.2 ± 0.02	1.4 ± 0.07
T_max_ (min)	15^*^	60^*^	15	15	15	30
AUC_(0-120min)_ %ID/g.min	195.3 ± 22.5^*^	100.2 ± 12.3^*^	31 ± 9.6	125.25 ± 21.3	7.1 ± 3.1	77.6 ± 15.3
Brain/Blood Ratio	1.9^*^		0.24		0.091	
LogBB at 15 min	0.7782		−0.556		−0.865	
LogBB at 120 min	0.2329		−0.477		−1.301

As perceived from Fig. 6A and B, which elucidates the uptake of radiolabelled free ZNS and ND-ZNS complex, oral and IN free ZNS had minimal distribution in the brain and higher distribution in the liver, kidney, and stomach. On the contrary, IN ND-ZNS complex F1 demonstrated a significantly higher concentration of ZNS in the brain and lower biodistribution in these organs with the least accumulation in the kidney in all collected time points (Fig. 6C) which is crucial for minimizing the risk of renal lithiasis [9]. These results confirm the failure of oral and IN free ZNS to attain sufficient therapeutic levels in the brain and suggest the superiority of the developed IN ND complex in delivering ZNS successfully to the brain.

Delving deeper into the distribution of ZNS across various tissues, it is observed that the drug is predominantly present in specific organs. ZNS accumulates in the liver and is primarily metabolized by cytochrome P450 and in the kidneys, where its metabolites and the non-metabolised dose fraction are excreted [4, 74]. The high ZNS affinity for erythrocytes is responsible for its appearance in the blood, while its detection in the stomach, even following IN administration, is due to mucociliary clearance and the escape of the non-absorbed portion of the dose into the stomach or lungs [75].

The careful inspection of the compared pharmacokinetic behaviour in blood and brain for the different treatments (Fig. 7) endorsed remarkable differences between the two routes of administration (oral and intranasal) and between IN free ZNS and IN ND-ZNS complex. In general, IN route offered a better ZNS distribution in the brain expressed by higher C_max_ of 0.5 ± 0.2% injected dose/gram organ weight (%ID/g of the brain) after 15 minutes and greater extent of ZNS absorption expressed by AUC _(0-120min)_ of 31±9.6 %IDg.min for IN free ZNS which is three and fivefold higher than the C_max_ and AUC _(0-120min)_ of oral free ZNS route of 0.2±0.02%ID/g of the brain and (7.1±3.1 %ID/g.min) respectively. However, it is interesting to point out the remarkable higher brain C_max_ of 3.9±0.1 %ID/g of the brain achieved by IN ND-ZNS treatment in 15 minutes, followed by a slow decline phase. This behaviour enabled the extension of ZNS presence in the brain in a concentration of 1.1±0.1 %ID/g of the brain after 2 hours, which is helpful for chronic epilepsy management [9]. The depicted slow decline in ZNS levels after IN ND-ZNS administration could be understood if one considers the in vivo stability of ND complexes due to their electrostatic solid interactions with ZNS and their ability to retain drugs for a longer time, thereby prolonging their efficacy and avoid frequent dosing [12, 76]. Moreover, It is undeniable that a significant (p≤0.05) overall enhanced pharmacokinetics expressed by AUC _(0-120min)_ of 195.3±22.6 %ID/g.min was perceived, which was 27 and 6-fold greater than free oral and IN free ZNS treatments of (7.1±3.1 and 31±9.6 %ID/g.min) respectively. This is directly linked with evident preferential brain uptake and a higher brain/blood ratio of 1.9 for IN ND-ZNS treatment compared to free ZNS oral treatment (0.09) and free ZNS IN treatment (0.24), confirming the added benefits of ND in the IN brain targeting. In addition, the role of ND in enhancing the brain accumulation and permeability of IN was confirmed by LogBB, which indicates high permeability and brain accumulation with values above 0.3 and poor brain accumulation with negative values below −1 [52, 77]. IN ND-ZNS showed high brain permeability with positive values of 0.77±0.08 at 15 minutes, in contrast to negative values of IN ZNS of −0.55±0.06 simultaneously, indicating poor brain permeation of IN free ZNS (Table 4).

Many scenarios could describe the superiority of the IN ZNS-ND route for ZNS brain targeting. First is the IN-route’s ability to bypass the BBB, offering better-targeted delivery of hydrophilic ZNS to the brain through the olfactory and trigeminal routes. On the contrary, in the oral route, ZNS is primarily absorbed into the bloodstream, leading to higher systemic exposure and causing the previously discussed side effects, with poor brain targeting due to the difficulties of hydrophilic ZNS passing through the BBB.

However, the complexation of ZNS with NDs augmented its IN delivery significantly, leading to its higher brain localization and minimal systemic distribution. The possible explanation for the observed results stems from the nano-size characteristics of the developed ZNS-ND complex F1, which facilitated the transcellular and paracellular passage through the olfactory and trigeminal routes directly to the brain through passive diffusion as well as endocytosis, therefore improving ZNS uptake, release, and bioavailability in the brain [8, 78, 79]. Successful endocytosis of nanodiamonds has been previously reported in the literature and attributed to receptor-mediated endocytosis through clathrin-coated pits along olfactory/trigeminal nerves to reach the CNS, avoiding systemic circulation [79]. In addition, their small size shows superior mucus penetration due to reduced steric hindrance with mucin fibers with pore sizes ~100–500 nm [80]. Moreover, the successful ND complexation imparts hydrophobic properties to the hydrophilic ZNS, allowing a fraction of ND-ZNS complex to partition into the lipid bilayer of the nasal epithelial cell membrane and is absorbed into the systemic circulation, which directly crosses the lipophilic BBB via transcellular diffusion due to the acquired lipophilicity [22, 81] thereby attaining high brain distribution.

Thus, keeping in mind that the currently marketed oral ZNS is extensively metabolized in the liver with common gastrointestinal and kidney lithiasis side effects therefore, without a doubt, the combined mechanisms offered by ND hold great promise as a drug carrier for delivering centrally acting ZNS not only intranasally, but also across the BBB with better tolerance and improved adverse effect. Nevertheless, the pharmacodynamics study will further confirm the potential role of NDs in the successful IN delivery of ZNS.

### In vivo anti-epileptic activity of intranasal ND-ZNS complex

The anti-epileptic activity following IN delivery of both ND-ZNS complex and free ZNS was studied to elucidate the added benefits of ND as IN drug delivery systems and their contribution to successful brain targeting in an experimental model of TLE in rats. Temporal lobe epilepsy (TLE) is the most common form of focal epilepsy in which seizures start in one or both temporal lobes of the brain [82]. The use of an experimental model that is aligned with the clinical features of human epilepsies is a vital process for investigating the effect of anti-epileptic drug formulations. The pilocarpine rodent model of epilepsy can exhibit seizures that evolve spontaneously following a post-insult latent period or arise within a developmental time frame consistent with the human condition of TLE [83]. Animals with the pilocarpine model of epilepsy show both the typical histopathological alterations and spontaneous chronic seizures seen in patients with TLE [49]. Therefore, this model was used in the current study to investigate the anti-epileptic activity of ND-ZNS IN complex compared to free IN drug.

#### The electroencephalography (EEG) and the behavioural seizures

**Fig. 8:**
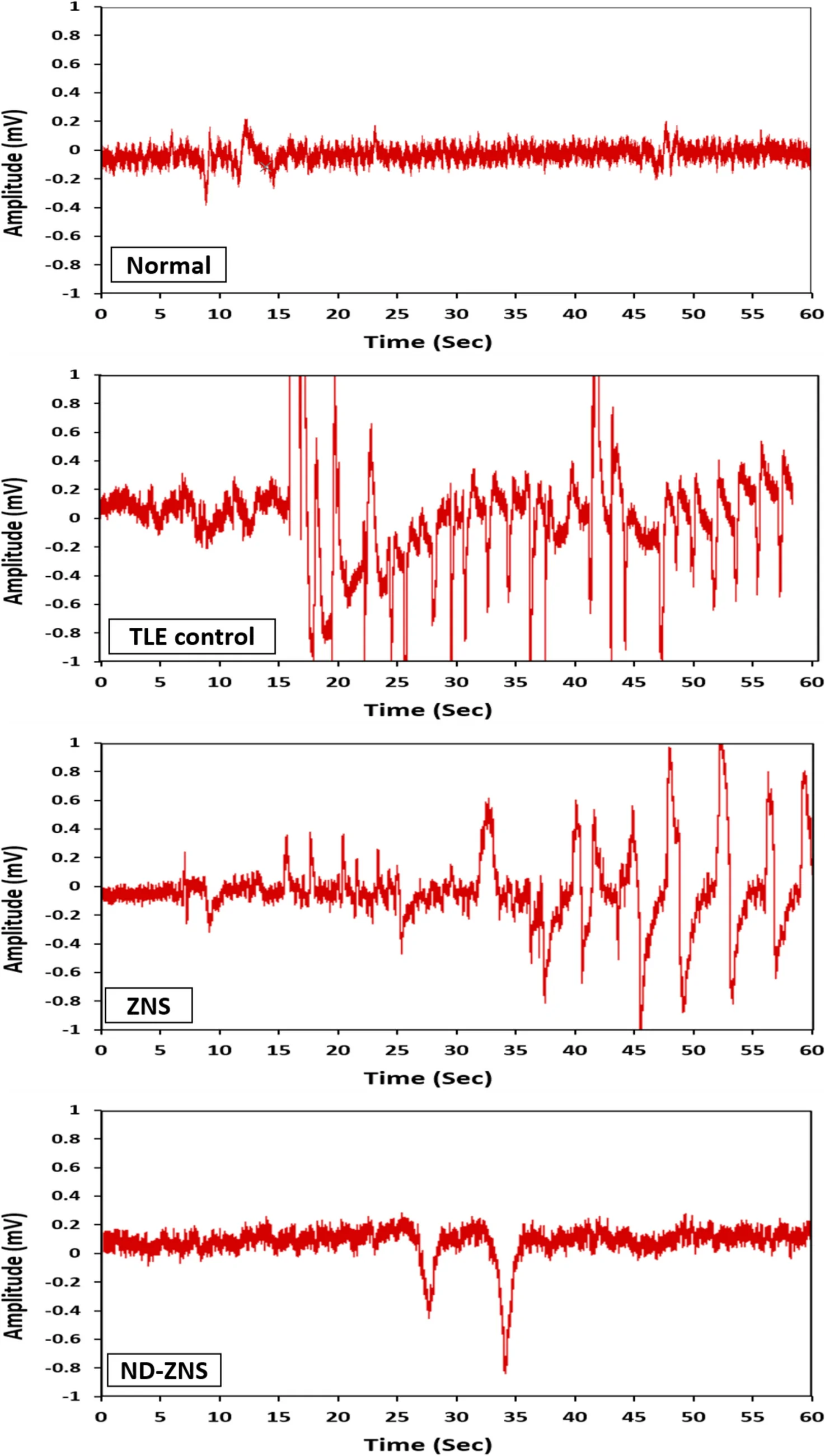
Effect of IN delivery of both IN ND-ZNS F1 complex and IN free ZNS in experimentally induced TLE in rats on the EEG recording ((*n* = 8, results presented as mean ± SEM), Significance level: *p < 0.05, **p < 0.01, ***p < 0.001)

**Fig. 9:**
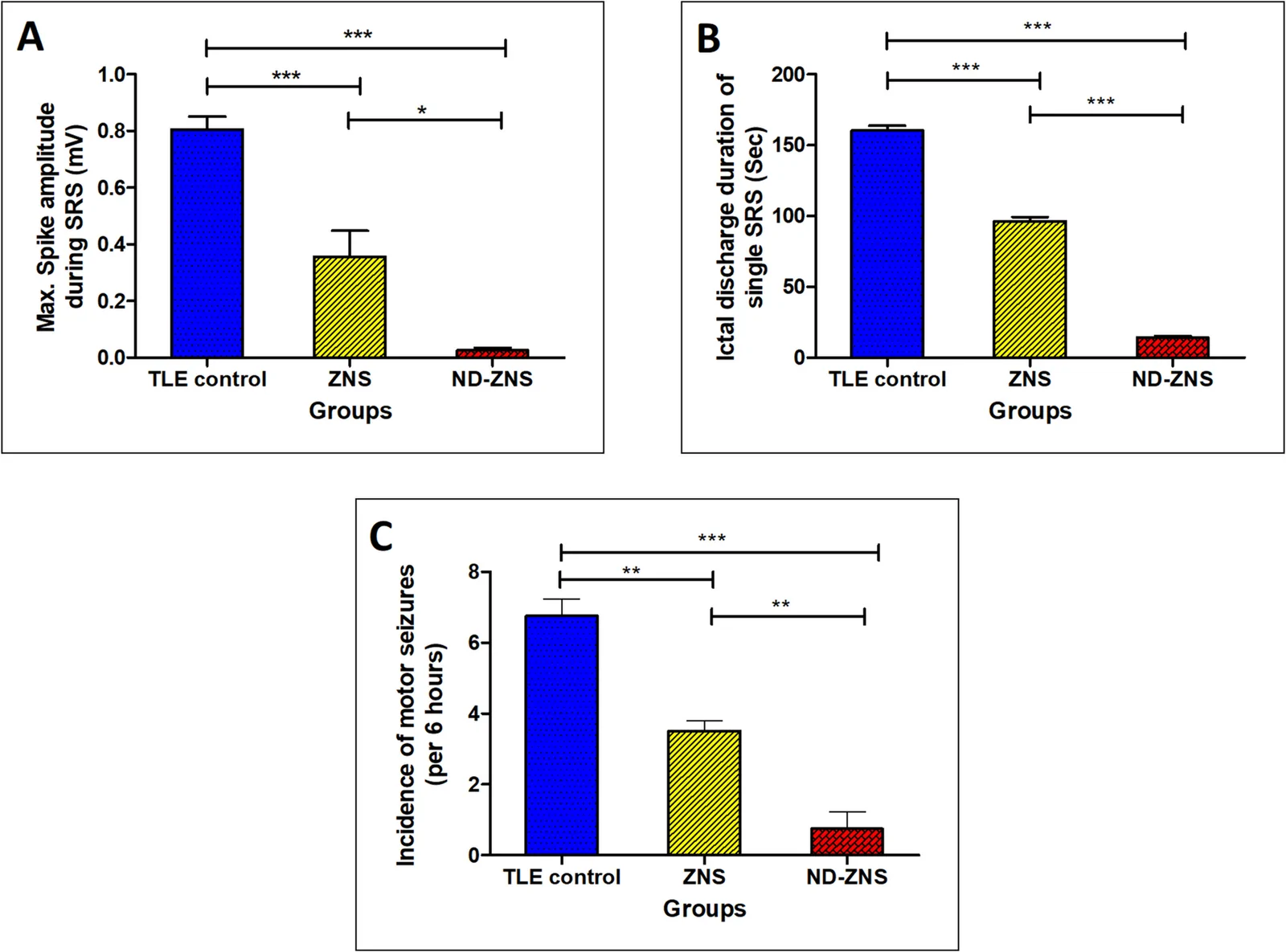
Effect of IN delivery of both IN ND-ZNS F1 complex and IN free ZNS in experimentally induced TLE in rats on EEG assessment and behavioural seizure incidence: Maximum spike amplitude (mV) during the SRS and Epileptogenic ictal discharge duration of single SRS in EEG and Incidence of motor seizures per 6 h, ((*n* = 8, results presented as mean ± SEM), Significance level: *p < 0.05, **p < 0.01, ***p < 0.001)

Epilepsy studies rely primarily on electroencephalography (EEG) signals to recognize, examine, and evaluate abnormal brain activity during seizures [84]. Several studies highlighted the role of EEG in diagnosing and monitoring TLE by recording the ictal discharges on the EEG, either clinically or in the experimental animal models of TLE [85–87]. Thus, EEG was used in the current study to evaluate brain activity following induction of TLE and treatment with IN ZNS (Figs. 8-9). Induction of TLE was demonstrated through the incidence of abnormal SRS characterized by epileptogenic ictal spikes discharge in EEG (mean duration of single SRS 160.4 ± 5.7 Sec and maximum spike amplitude of 0.8 ± 0.04 mV. In addition, video recordings for 6 hours per day confirmed an incidence of motor seizures in the untreated TLE group of 6.8 ± 0.4 incidents, with seizure scores ranging from 2 to 3 based on the Racine scoring system (Fig. 9). Upon treatment with IN free ZNS, a significant decrease in the duration and amplitude of the SRS (mean duration of single SRS of 96.5 ± 2.9 Sec. and max. spike amplitude of 0.4 ± 0.1 mV) with a significant decrease in the incidence number of the motor seizures (3.5 ± 0.5) when compared with the TLE control rats and without a change in seizures scoring number that ranged from 2 to 3 (Figs. 8-9).

Nevertheless, treatment with IN ND-ZNS complex F1 markedly improved the EEG and revealed almost normal EEG patterns with few downward spikes in some instances (mean duration of single SRS 14.2 ± 1.2 Sec. and max. spike amplitude of 0.03 ± 0.01 mV). Moreover, IN ND-ZNS treatment significantly decreased the incidence of motor seizures (0.8 ± 0.4), with Racine scoring ranging from 1 to 2. These results align with the biodistribution results confirming the role of ND in successfully delivering ZNS to the brain with sufficient concentration for efficacious treatment of epilepsy.

### Serum levels of NSE, NEFL, and MMP-9

**Fig. 10:**
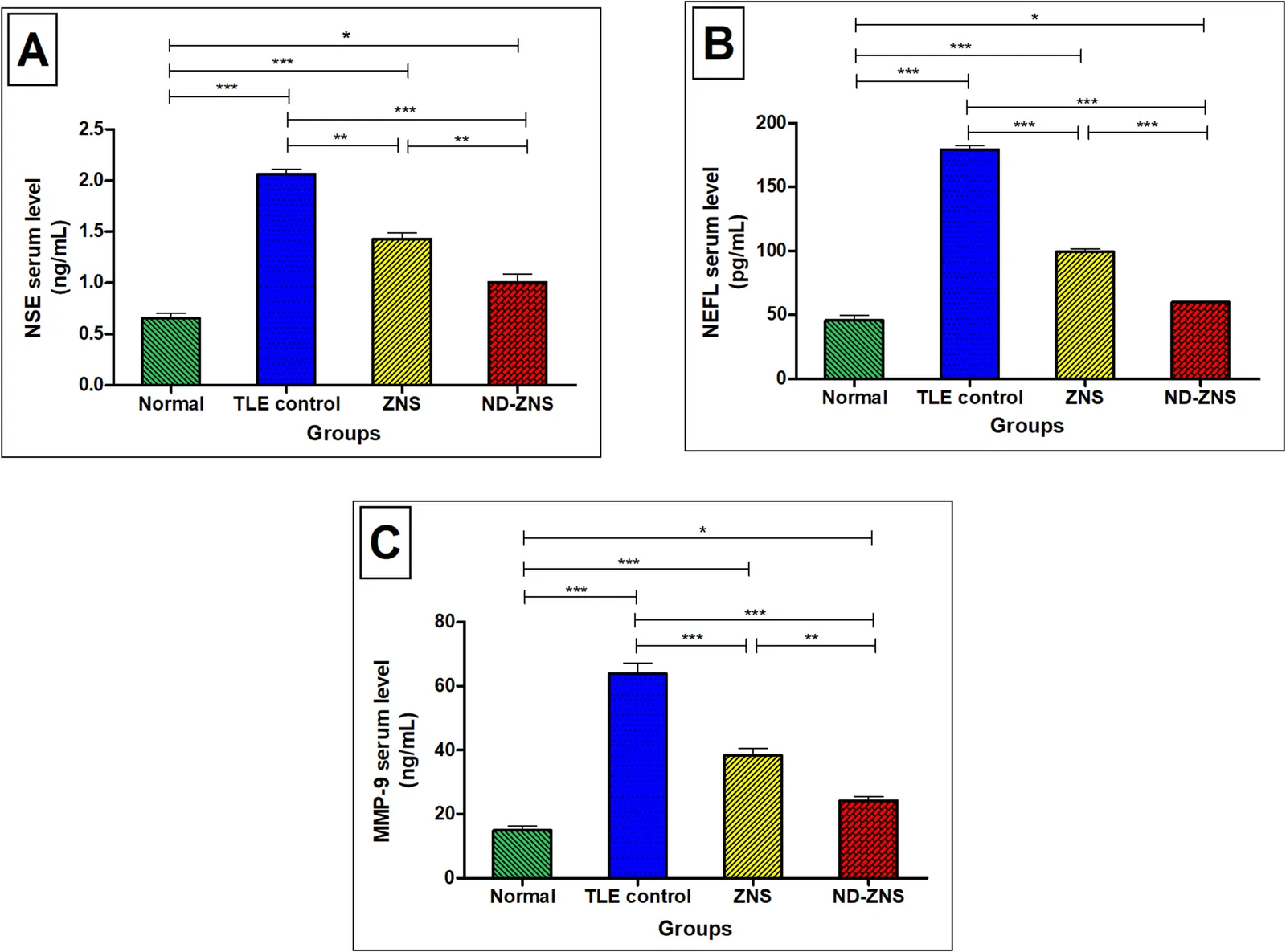
Effect of IN delivery of both IN ND-ZNS complex and free IN ZNS on serum levels of (**A**) NSE, (**B**) NEFL and (**C**) MMP-9 in experimentally induced TLE in rats. (*n* = 8, results presented as mean ± SEM, Significance level: *p < 0.05, **p < 0.01, ***p < 0.001)

Robust and accessible diagnostic and prognostic biomarkers are greatly needed in epilepsy. Epilepsy diagnosis is most often based on symptom descriptions, and misdiagnosis of epilepsy can bring substantial human and financial costs [88]. Recently, some biochemical markers were reported to have higher blood concentrations in study subjects with epilepsy, including brain neuronal specific enolase (NSE), neurofilament light polypeptide (NEFL), and matrix metallopeptidase-9 (MMP-9) that can detect the pathophysiological changes associated with epilepsy and reflect seizures duration and frequency. The decrease in their blood levels was directly correlated with anti-seizure treatment [89]. NSE is a glycolytic enzyme unique to neurons and neuroendocrine cells that has been shown to increase even after a single seizure [90, 91] and reflects seizure frequency in TLE [92, 93]. NEFL is the axonal proteins that maintain the structure of neurons and are subsequently released into the cerebrospinal fluid and bloodstream upon neuroaxonal damage after epileptic seizures [94–96]. MMP-9 is a proteolytic enzyme suggested to play a role in epileptic focus formation and the stimulation of TLE seizures [97, 98]. A previous study has demonstrated the correlations of epileptic seizure severity and frequency with the serum level of MMP-9 [99]. Thus, biochemical analysis of these biomarkers in rats was performed to compare the efficacy of IN free ZNS solution to IN ND-ZNS complex. As shown in Fig. 10, a significant increase in the serum levels of NSE, NEFL, and MMP-9 in the TLE control group (by 3.1, 3.9, and 4.3 folds, respectively) was observed when compared to the normal rats (*p* < 0.001) confirming successful induction and correlation of these biomarkers with epilepsy.

Moreover, there was a significant decrease in the serum levels of NSE, NEFL, and MMP-9 by 30.6%, 44.6%, and 40.1%, respectively, in the ZNS solution-treated group compared to the TLE control rats. On the other hand, treatment with the ND-ZNS formulation resulted in a significantly higher suppression in the serum levels of NSE, NEFL, and MMP-9 by 51.2%, 66.6%, and 62.1%, respectively, when compared to the TLE control and the ZNS solution treated groups. The ND-ZNS group showed a better significant effect than the ZNS group (*p* < 0.01) with just a slight difference from the normal group (*p* < 0.05), as shown in Fig. 10, providing robust evidence for the improved anti-seizure activity with the ND delivery system to almost normal conditions. These results corroborate the role of ND as a successful delivery system for intranasal ZNS to the brain more efficiently than plain ZNS solution to achieve better therapeutic outcomes.

#### Hippocampal expression levels of miRNA-199, SIRT-1, LncRNA-PVT1, and serum level of BDNF

At the molecular level, seizure activity has been shown to mediate epigenetic changes, including the recruitment of non-coding RNAs (ncRNAs) that induce alterations in the expression of genes controlling neurotransmitter signaling, ion channels, synaptic structure, neuronal death, and inflammation [100]. Long non-coding RNA (LncRNA)-Plasmacytoma variant translocation 1 (PVT1), which negatively regulates the expression of the neuroprotective brain-derived neurotrophic factor (BDNF), was reported to exhibit high expression in the epileptic rat hippocampus and increase neuronal cell loss [101]. Moreover, BDNF controls the growth of axons and synaptogenesis and reverts many histological alterations associated with chronic epilepsy. Thus, both biomarkers can be used for assessing brain damage in Epilepsy [51]. Moreover, micro-RNA-199 (MiR-199), a small non-coding RNA, has been reported to play a vital role in the regulation of epileptic seizures by targeting the mRNA of the antiapoptotic protein Silent Information Regulator 1 (SIRT1) and inhibiting its expression levels [102]. The upregulation of the hippocampal expression level of miR-199 can further induce apoptosis and neuron loss in epilepsy [50]. Therefore, miR-199/SIRT1 can represent an important regulatory pathway in epilepsy.

**Fig. 11:**
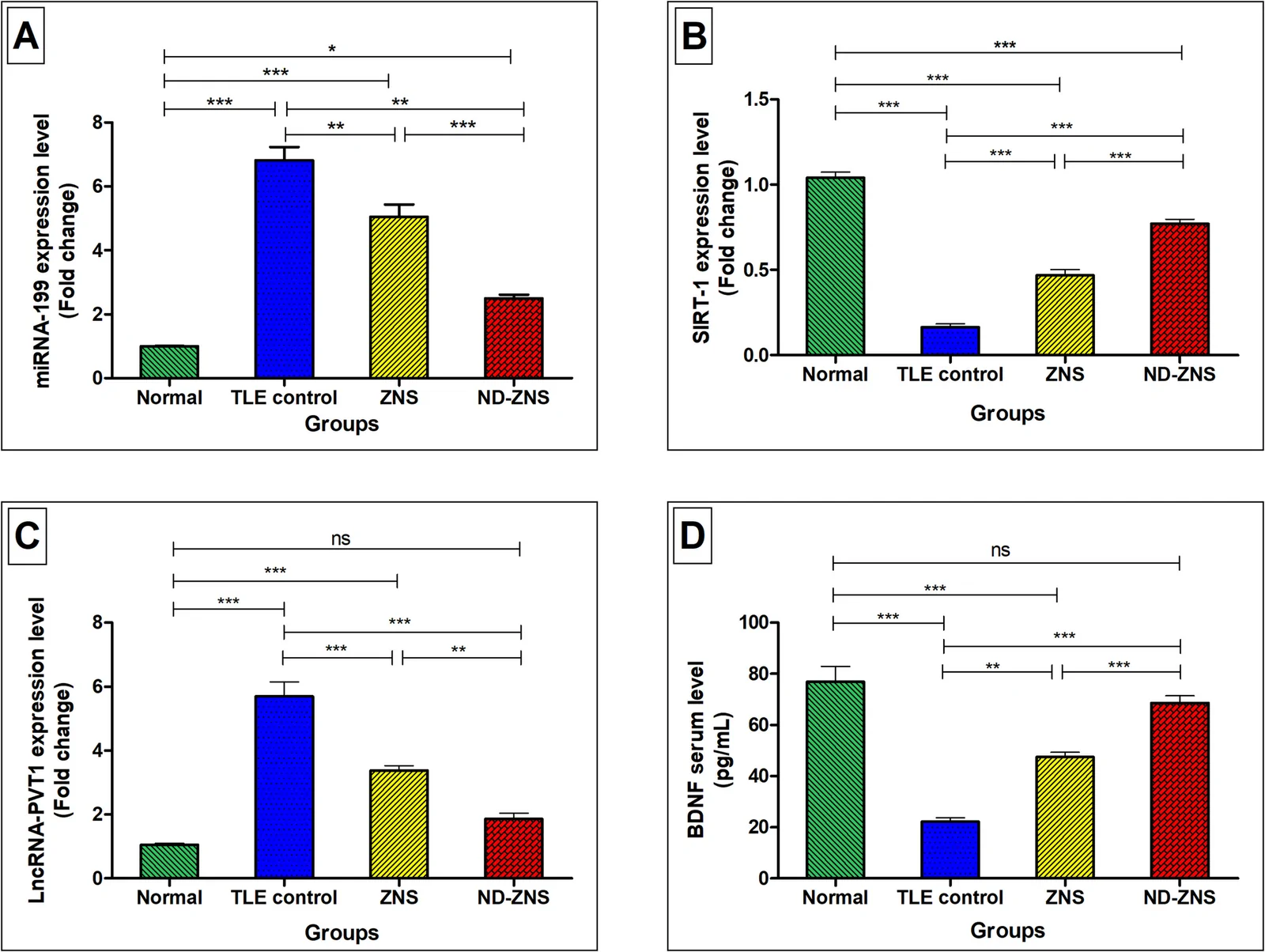
Hippocampal expression levels of (**A**) miRNA-199, (**B**) SIRT-1, (**C**) LncRNA-PVT1, and (**D**) serum level of brain-derived neurotrophic factor (BDNF) in experimentally induced TLE in rats. (*n* = 8, results presented as mean ± SEM, Significance level: *p < 0.05, **p < 0.01, ***p < 0.001)

Epigenetic mechanisms were recently demonstrated to play a vital role in epileptogenesis through the modulation of many critical biological pathways that can be regulated by microRNAs (miRNA) and long non-coding RNAs (LncRNA) ([103, 104]. MiR-199 is reported to be explicitly expressed in neural tissues, such as the hippocampus, and associated with regulating seizures and seizure damage by targeting and inhibiting the antiapoptotic protein silent information regulator-1 (SIRT-1) [50, 105]. Investigation of miR-199/SIRT-1 pathway in the current study showed a significant increase in the hippocampal expression of miR-199 by 6.8 folds in the TLE control group when compared to the normal rats *(p* < 0.001) accompanied by a marked suppression of SIRT-1 in the TLE control group, that was only 0.16 fold of the normal SIRT-1 level in hippocampus. The current results are consistent with other studies that highlighted the role of the miR-199/SIRT-1 axis in epilepsy through inhibiting SIRT-1 as a crucial endogenous apoptosis inhibitor that can promote mammalian axon regeneration [50]. Treatment with IN ZNS solution resulted in a significant decrease in the hippocampal level of miRNA-199 (5.05 folds) and a significant increase in the SIRT-1 (0.47 folds) compared to the TLE control group. Furthermore, IN ND-ZNS treatment resulted in better improvement with marked suppression of the hippocampal levels of miRNA-199 (2.5 folds) and elevation of the SIRT-1 level (0.77 folds) with a noticeable significant difference from the ZNS solution-treated group (*P* < 0.001) (Fig. 11). The results of this study suggest that ZNS exerts a seizure-suppressing effect in rats through inhibition of miR-199 and upregulation of its direct target SIRT-1 in the hippocampus. In addition, these results further support the marked improvement in the anti-seizure activity of the ND-ZNS formulation compared to the ZNS solution through modulating the miR-199/SIRT-1 pathway that represents a potential target for preventing and treating epilepsy.

Furthermore, the current study investigated the role of LncRNA-PVT1 in epileptogenesis by targeting brain-derived neurotrophic factor (BDNF). The TLE control group showed a significant elevation in the hippocampal expression of LncRNA-PVT1 by 5.7 folds compared to the normal rats *(p* < 0.001). These results are accompanied by a marked suppression of the serum level of BDNF (22.1 ± 1.7 pg/mL) in the TLE control group compared to the normal group (76.8 ± 6.1 pg/mL). These results are in harmony with another study that reported the overexpression of lncRNA-PVT-1 in the hippocampus of epileptic rats with marked neuronal loss through suppressing the expression of BDNF, resulting in further inhibition of neurogenesis and axonal development [106]. Treatment with ZNS solution resulted in a significant decrease in the hippocampal level of LncRNA-PVT1 (3.4 folds) and a significant increase in BDNF (47.45 ± 1.9 pg/mL) compared to the TLE control group. On the other hand, ND-ZNS treatment resulted in the normalization of the hippocampal expression of LncRNA-PVT1 and the serum level of BDNF with a non-significant difference from the normal group reflecting the significant role of the ND formulation in improving the anti-epileptic activity of ZNS (Fig. 11). The upregulation of BDNF was previously reported to provide a protective response against seizures through counteracting the hippocampal epileptogenesis [107].

#### Histopathological examination of the brain hippocampus

**Fig. 12:**
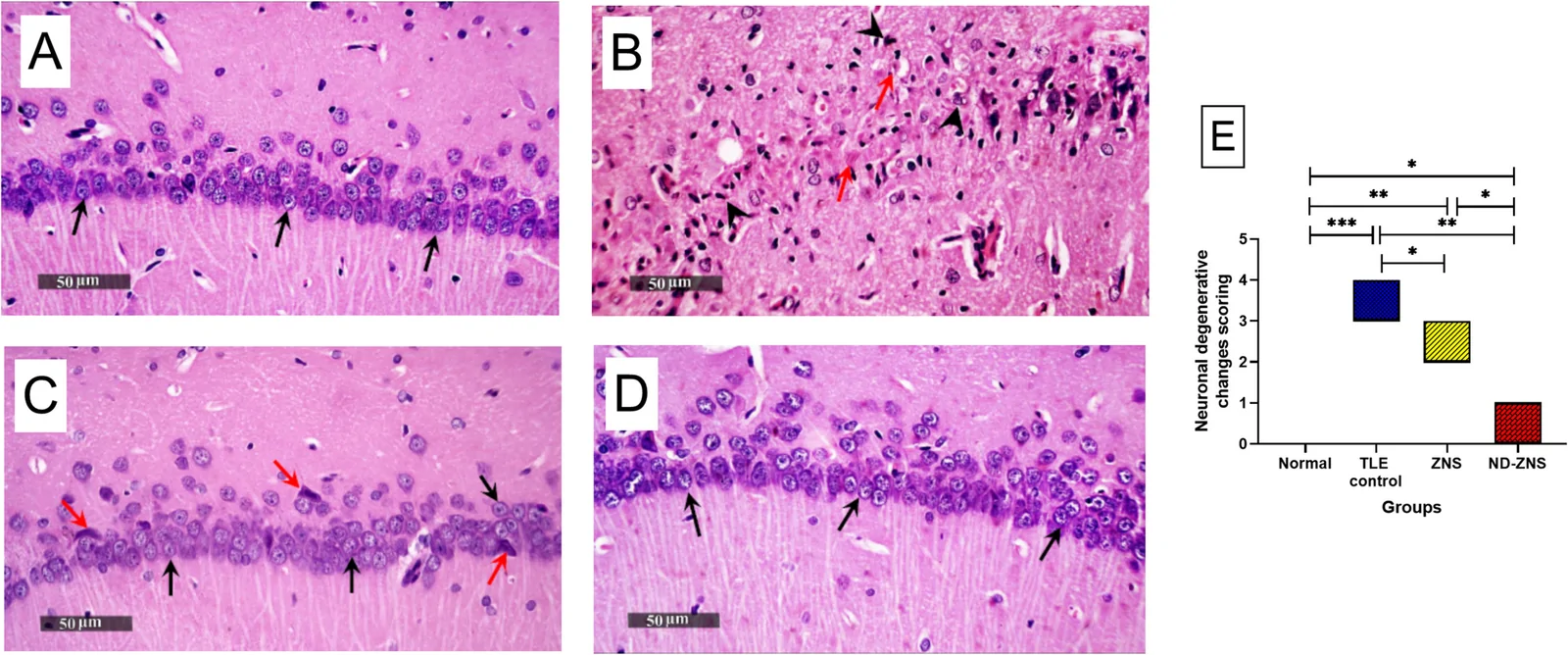
Histopathological examination of brain hippocampus in experimentally induced TLE in rats: [**A**] Normal group, [**B**] TLE control group, [**C**] free IN ZNS treated group, [**D**] IN ND-ZNS F1 treated group, [**E**] Scoring of neuronal degenerative changes in rats hippocampi among the different groups. Data are expressed as box plots of the median. Significance level: *p < 0.05, **p < 0.01, ***p < 0.001using the Kruskal–Wallis test followed by Dunn’s test

Histopathological examination of hippocampal brain samples from the different groups has further supported the study findings. The normal control samples showed normal organized histological features of hippocampal layers, including pyramidal neurons with intact nuclear and cytoplasmic details (black arrow) and an intact intercellular brain matrix without abnormal cellular infiltrates (Fig. 12A). The TLE control group showed severe neuronal damage with abundant regions of necrotic pyramidal neurons (red arrow) associated with reactive microglial infiltrates (arrowhead) (Fig. 12B). The ZNS-treated group demonstrated noticeable neuroprotective efficacy, with mildly scattered figures of persistent neuronal damage (red arrow) alternated with many apparent intact neurons with intact subcellular details (black arrow) along with a few reactive glial cells observed (Fig. 121). Treatment with the ND-ZNS formulation resulted in a greater degree of neuroprotective efficacy, with almost intact, well organized morphological features of CA1 subregions of the hippocampus displaying many apparent intact pyramidal neurons (black arrow) and minimal sporadic records of neuronal damage (red arrow) (Fig. 12D). These results were confirmed by Scoring of neuronal degenerative changes in rats hippocampi among the different groups which showed the highest damage in the untreated group, while treatment with IN ND-ZNS resulted in a significantly decreased neurodegenerative damage score (*p*>0.05) (Fig. 12E).

## Conclusion

An original, simple nose-to-brain ND-ZNS delivery system was successfully developed and optimized using computational software applications. ND-ZNS complex offered a superior brain targeting of ZNS in a biphasic release pattern to rapidly achieve high ZNS concentrations, followed by a sustained pattern that can be used in acute and chronic epilepsy management with less frequent administration. Limited systemic peripheral exposure was confirmed by the in vivo biodistribution and pharmacokinetics studies, which guard against drug-resistant epilepsy and side effects associated with currently marketed oral ZNS. Furthermore, results of the in vivo effects of IN ND-ZNS formulation revealed enhanced brain targeting and localization compared to both oral and IN free ZNS with marked improvement in the anti-epileptic activity with a significantly improved EEG pattern, epileptic biomarkers and hippocampal histological structure in the experimentally induced TLE model. IN ND-ZNS formulation also significantly suppressed the miR-199/SIRT-1 pathway and normalized the PVT-1/BDNF pathway in the TLE model. Indeed, NTB delivery of ND-ZNS holds promise as a novel, potential, tolerable approach for effective ZNS brain targeting in epilepsy management.

## Data Availability

The authors confirm that the data supporting this study's findings are available within the article. The corresponding author can share the data upon reasonable request.
